# The N-terminus of an *Ustilaginoidea virens* Ser-Thr-rich glycosylphosphatidylinositol-anchored protein elicits plant immunity as a MAMP

**DOI:** 10.1038/s41467-021-22660-9

**Published:** 2021-04-27

**Authors:** Tianqiao Song, You Zhang, Qi Zhang, Xiong Zhang, Danyu Shen, Junjie Yu, Mina Yu, Xiayan Pan, Huijuan Cao, Mingli Yong, Zhongqiang Qi, Yan Du, Rongsheng Zhang, Xiaole Yin, Junqing Qiao, Youzhou Liu, Wende Liu, Wenxian Sun, Zhengguang Zhang, Yuanchao Wang, Daolong Dou, Zhenchuan Ma, Yongfeng Liu

**Affiliations:** 1 Institute of Plant Protection, Jiangsu Academy of Agricultural Sciences, Nanjing, China; 2 Department of Plant Pathology, Nanjing Agricultural University, Nanjing, China; 3 State Laboratory for Biology of Plant Diseases and Insect Pests, Institute of Plant Protection, Chinese Academy of Agricultural Sciences, Beijing, China; 4 College of Plant Protection, Jilin Agricultural University, Changchun, China

**Keywords:** Effectors in plant pathology, Microbe, Virulence

## Abstract

Many pathogens infect hosts through specific organs, such as *Ustilaginoidea virens*, which infects rice panicles. Here, we show that a microbe-associated molecular pattern (MAMP), Ser-Thr-rich Glycosyl-phosphatidyl-inositol-anchored protein (SGP1) from *U. virens*, induces immune responses in rice leaves but not panicles. SGP1 is widely distributed among fungi and acts as a proteinaceous, thermostable elicitor of BAK1-dependent defense responses in *N. benthamiana*. Plants specifically recognize a 22 amino acid peptide (SGP1 N terminus peptide 22, SNP22) in its N-terminus that induces cell death, oxidative burst, and defense-related gene expression. Exposure to SNP22 enhances rice immunity signaling and resistance to infection by multiple fungal and bacterial pathogens. Interestingly, while SGP1 can activate immune responses in leaves, SGP1 is required for *U. virens* infection of rice panicles in vivo, showing it contributes to the virulence of a panicle adapted pathogen.

## Abstract (web summary)

*Ustilaginoidea virens* is a fungal pathogen that infects rice via the panicles. Here, the authors show that *U. virens* SGP1, a conserved Ser-Thr-rich glycosyl-phosphatidyl-inositol-anchored protein, elicits immune responses in rice leaves while contributing to virulence in panicles.

## Introduction

The ability to sense the presence of potentially infectious pathogens poses a central challenge for all multicellular organisms and forms the basis for activation of innate defense mechanisms against attempted microbial infection^1^. Plant perception of molecular determinants, known as microbe-associated molecular patterns (MAMPs) or pathogen-associated molecular patterns (PAMPs)^1–3^, which are conserved and characteristic of classes of microbes, can trigger basal immune responses^4,5^.

MAMPs/PAMPs have been described for bacteria, oomycetes, and fungi. They can be peptidoglycans^6^, lipopolysaccharides^7^, glucans^8^, chitins^9^, or proteins encoded for diverse functions. Well-characterized proteinaceous MAMPs/PAMPs include flagellin^10^, EF-Tu^11,12^, and cold shock proteins (CSPs)^13^ from bacteria, transglutaminases (TGases)^14^, cellulose-binding elicitor lectin (CBEL)^15^, and elicitins^16^ from oomycetes, and xylanase EIX^17^ from fungi. The necrosis and ethylene-inducing peptide 1 (Nep1)-like proteins (NLPs)^18–22^ and glycoside hydrolase 12 protein XEG1^23,24^ MAMPs are both extensively distributed in bacteria, oomycetes, and fungi. Typically, small epitopes within the microbial patterns are sufficient to elicit immunogenic responses^4,25,26^. For example, a highly conserved 22-aa fragment of flagellin, flg22, is sufficient to activate immunity in *Arabidopsis* and other plant species^10^. An 18-aa domain of EF-Tu, elf18, is recognized as a MAMP in Brassicaceae species^11,12^. Pep‐13, which is conserved among *Phytophthora* TGases, activates defense reactions in parsley and potato^14^. However, few peptides from proteinaceous MAMPs have been identified in fungi, with the exception of 24-aa peptides of NLPs that act as MAMPs in *Arabidopsis*^19^. Thus, the mechanisms that allow plants to perceive pathogenic fungi and to defend against infection remain mostly undiscovered.

Glycosyl-phosphatidyl-inositol (GPI)-anchored proteins (GAPs) are a class of membrane-associated proteins containing a soluble protein attached by a post-translational glycolipid modification, the GPI anchor, to the external leaflet of the plasma membrane^27^. GPI is broadly distributed among eukaryotes, including protozoa, fungi, plants, insects, and mammals, while proteins containing a GPI anchor are structurally and functionally diverse and play vital roles in numerous biological processes, as enzymes, cell surface antigens, signaling receptors, cell adhesion molecules, and migration molecules^28,29^. For example, the GPI-anchored proteins CD55 (or decay-accelerating factor, DAF) and CD59 are important in the regulation of the complement cascade, which protects organisms from foreign invaders and pathogens^30^. Phospholipases (PLCs) can cleave the GPI anchor and release GAPs^31^. Approximately 1% of protein-coding genes in eukaryotic genomes are likely to encode GPI-anchored proteins (GAPs)^32^. Nevertheless, the specific roles of the majority of GAPs in host–microbe interactions remain largely unknown, especially in plant pathogens.

*Ustilaginoidea virens* (Cooke) Takah is the causal agent of rice false smut (RFS), which has recently undergone a rapid expansion to emerge as one of the most destructive rice diseases worldwide^33,34^. For example, in China, an estimated ~2.4 million hectares per year between 2015 and 2017 are reportedly affected by this disease^35^. RFS epidemics not only lead to yield loss but also threaten food safety due to its production of mycotoxins, including ustiloxins and ustilaginoidins^35^. This pathogen is an ascomycetous fungus that predominantly attacks rice stamen filaments, extending intercellularly along the filament base, and sometimes invades lodicules^33,36,37^, but is asymptomatic in other organs such as leaves, thus leading to its categorization as a new type of flower disease^38^. The most likely route of *U. virens* infection is entry into the inner space of the spikelets through the gap between the lemma and palea when rice plants are at the booting stage^39,40^. Given its recent emergence, the mechanisms underlying plant–*U. virens* interactions and how plants defend themselves against infection by this pathogen remain largely unknown.

Here, we identified a MAMP, SGP1, from the causal agent of RFS, *U. virens*, that triggers programmed cell death in several plant species, including *N. benthamiana* in a BAK1-dependent manner. SGP1 belongs to the Ser-Thr-rich GPI-anchored protein family, which is widely distributed in fungi, and many homologs of which induce cell death in *N. benthamiana*. Plants specifically recognize a peptide comprising 22 amino acids (SGP1 N terminus peptide 22, SNP22) in the N terminus of the protein, which can fully activate programmed cell death, oxidative burst, and upregulation of defense-related gene expression, ultimately inducing resistance against both bacterial and fungal pathogens in rice. Notably, we show that SNP22 could elicit a stronger immunity signaling response in leaves than in panicles of rice. Moreover, using SGP1 overexpression lines, SGP1^W44A^ overexpression (with abolished SGP1-associated MAMP activity) lines, and SGP1-silenced lines, we found that SGP1 is required for *U. virens* pathogenicity during infection of rice panicles in vivo, and that it appears to activate an immune response in rice leaves.

## Results

### SGP1 from *U. virens* is an apoplastic elicitor of cell death in *N. benthamiana*

**Fig. 1:**
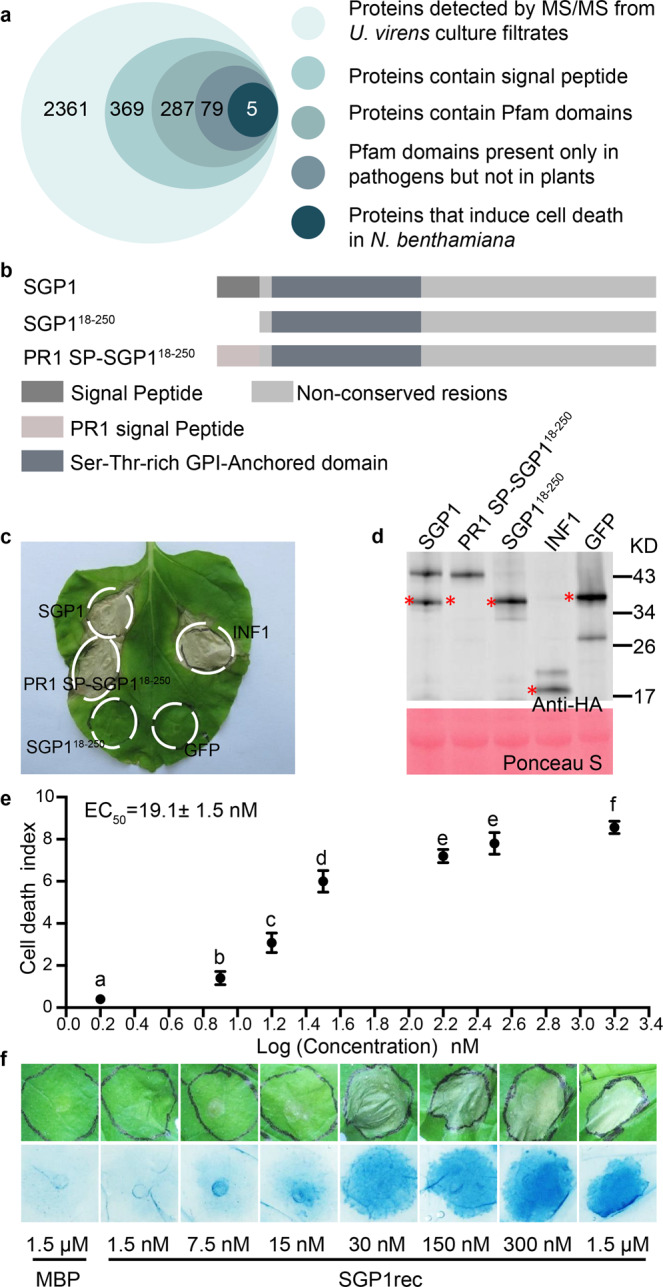
SGP1 is an apoplastic elicitor of cell death in *N. benthamiana*. **a** Systematic identification of SGP1 among *U. virens* secreted proteins. The numbers in circles represent proteins that met successively stringent criteria. **b** Regions of SGP1 examined for cell death activity. **c** Representative *N. benthamiana* leaves at 5 d after inoculation with *Agrobacterium* strains carrying the indicated genes in vector pGR107. Similar results were obtained from 3 independent experiments (Supplementary Data 3). **d** Western blot analysis of the indicated proteins transiently expressed in *N. benthamiana*. The red asterisks (*) indicate the predicted protein sizes in kDa. **e**, **f** Cell death induced by recombinant SGP1 protein from *E. coli*. MBP protein serves as a control (MBP, as the tag in empty vector, was purified in the same way as SGP1rec). Five independent experiments were performed, with five plants in each experiment. **e** Determination of purified SGP1rec EC_50_ values in cell death-inducing activity, based on cell death index (Supplementary Fig. 2). Values are means ± SD of five experiments. Different letters indicate statistically significant differences (*P* < 0.01; Duncan’s multiple range test). **f** Representative *N. benthamiana* leaves infiltrated with purified SGP1rec (1.5 nM to 1.5 μM). Upper panel, photos directly taken at 2 days post-infiltration (dpi). Lower panel, photos taken at 2 dpi after staining with trypan blue.

To identify potential elicitors of plant immunity from *U. virens*, we identified a library of 79 putative candidate proteins based on the following characteristics: 1. Proteins were detectable by MS/MS in *U. virens* culture filtrates from three independent experiments; 2. Proteins were predicted to contain a signal peptide by SignalP3.0; 3. Proteins were predicted to contain Pfam domains by Pfam 31.0; and 4. The predicted Pfam domains were present only in microbes, but not in plants (i.e., rice and *N. benthamiana*) (Fig. 1a and Supplementary Data 1). We then constructed an *Agrobacterium*-mediated transient expression system in *N. benthamiana* to assay the elicitor activity of candidates, which ultimately revealed five proteins that induced cell death in *N. benthamiana* leaves (Fig. 1a and Supplementary Data 1). A Ser-Thr-rich Glycosyl-phosphatidyl-inositol-anchored family protein (SGP1), which induced cell death in *N. benthamiana* and is widely distributed across fungi, was selected for further study (Fig. 1b–d and Supplementary Data 1–3). In addition, we identified a paralog of SGP1 in *U. virens,* which did not induce cell death in *N. benthamiana* (Supplementary Data 1 and 2).

In order to determine the role of the signal peptide in SGP1 activity, we deleted this region and found that loss of the signal peptide abolished the cell death-inducing ability of SGP1 in *N. benthamiana*. By contrast, replacing the signal peptide of SGP1 with the pathogenesis-related protein 1 (PR1) signal peptide, resulted in complementation of cell death-inducing activity (Fig. 1c and Supplementary Data 3). Western blot analysis showed an extra, larger band in addition to the bands at the predicted size for SGP1 and PR1 SP-SGP1^18–250^ but not at SGP1^18–250^ (Fig. 1d), suggesting that this band is likely a post-translationally modified form of SGP1. Together, these data suggested that SGP1 must be targeted to the extracellular space of *N. benthamiana* tissue to induce cell death.

### The SGP1 protein induces cell death in *N. benthamiana*, potato plants, and rice

To further confirm that SGP1 could induce cell death in *N. benthamiana*, the 68 kDa recombinant protein, SGP1rec (Supplementary Fig. 1), was infiltrated into the mesophyll of *N. benthamiana* leaves in a concentration from 1.5 nM to 1.5 μM. As shown in Fig. 1e, f and Supplementary Fig. 2, the degree of cell death increased with increasing concentrations of SGP1rec. The half-maximal effective concentration (EC_50_) value was calculated to be 19.1 nM (Fig. 1e, f).

**Fig. 2:**
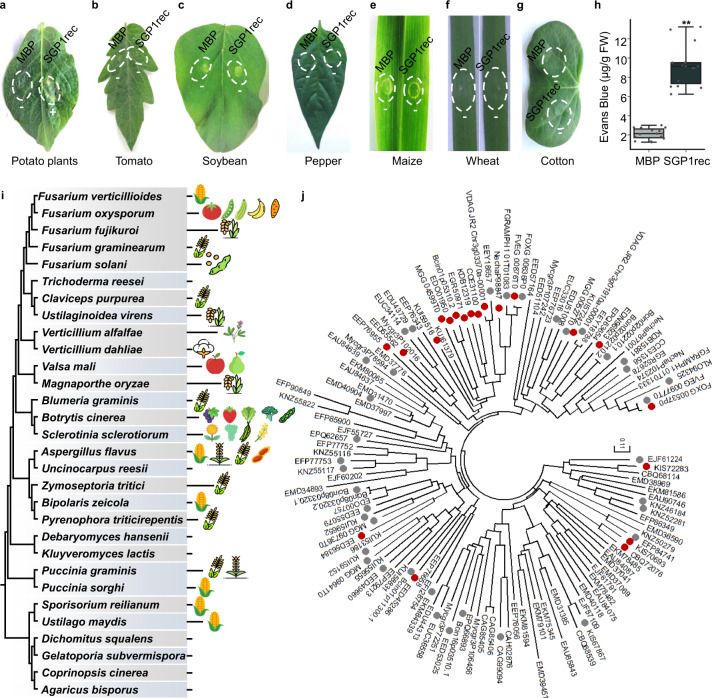
Specificity of the host response to SGP1 and wide distribution of SGP1 homologs across fungal species. **a**–**h** Cell death responses in other plant species triggered by exposure to 1 μM SGP1rec or MBP control. **a**–**g** Representative leaves are shown for potato plants (**a**), tomato (**b**), soybean (**c**), pepper (**d**), maize (**e**), wheat (**f**), and cotton (**g**). Data were obtained from three independent experiments, with 10 plants of each species per experiment (Supplementary Data 3). Dotted circles indicate infiltration area. The plus symbol (+) indicates that the cell death phenotype was observed, and the minus symbol (−) indicates the absence of a clear cell death phenotype. **h** Cell death induced by SGP1rec in rice suspension cells. Cell death was quantified by detection of Evans Blue signal released from rice suspension cells (μg/g) after treatment with SGP1rec or MBP. Data were obtained from five independent experiments, with three replicates in each experiment. In box plots, whiskers indicate the minimum and maximum values, the line indicates the median, the box boundaries indicate the upper (25th percentile) and lower (75th percentile) quartiles. **P < 0.01, two-sided *t*-test. **i** Distribution of SGP1 across fungal species. The fungal species include both plant pathogenic and non-pathogenic fungi. Left, Maximum-Likelihood phylogenetic tree of fungal species which contain the Ser-Thr-rich GPI-anchored domain, reconstructed using PhyML v3.0 based on single-copy orthologs (Supplementary Data 5). Right, representative hosts of the corresponding pathogens. Image credit: https://www.flaticon.com or https://icons8.com/icons. **j** Phylogeny of SGP1 and 123 related sequences from selected fungal species (Supplementary Data 2 and 4). The proteins that induce cell death in *N. benthamiana* are indicated by a red dot and those that do not are indicated by a gray dot.

To examine the specificity of the host response to SGP1, we infiltrated SGP1rec into expanded leaves of various plants. SGP1rec induced localized cell death in potato (*Solanum tuberosum*), but not in tomato (*Solanum lycopersicum*), soybean (*Glycine max*), pepper (*Capsicum annum*), maize (*Zea mays*), wheat (*Triticum aestivum*), and cotton (*Gossypium hirsutum*) (Fig. 2a–g and Supplementary Data 3). Due to technical issues with infiltration of SGP1rec into rice leaves, rice suspension cells were instead used to determine the cell death activity of SGP1 by Evans blue staining. As shown in Fig. 2h, exposure of cells to SGP1rec resulted in a threefold increase in Evans blue signal compared with controls at 12 h after treatment, thus indicating that SGP1rec induced cell death in rice suspension cells. Together, these experiments showed that SGP1 can induce cell death in at least three different plant species.

### SGP1 belongs to the Ser-Thr-rich Glycosyl-phosphatidyl-inositol-anchored protein family, which is widely distributed across fungi

Since SGP1 contains a distinctive Ser-Thr-rich GPI-anchored family domain, we studied the phylogenetic distribution of this protein domain in various organisms. We subsequently performed annotation of Pfam Ser-Thr-rich GPI-anchored domain (PF10342.8)-containing proteins in five plants, nine bacteria, five oomycetes, and thirty fungi (Supplementary Data 2). However, no proteins containing this domain were found in the plant, bacterial or oomycete species, but they were widely present in fungi, including crop pathogens such as *Puccinia graminis*, *Fusarium solani*, and *Magnaporthe oryzae*; vegetable pathogens such as *Botrytis cinerea* and *Sclerotinia sclerotiorum*; and fruit pathogens such as *Valsa mali* (Fig. 2i and Supplementary Data 2, 4, 5). These results suggested that Ser-Thr-rich GPI-anchored family proteins are common across fungal taxa.

The Ser-Thr-rich GPI-anchored family proteins from diverse fungal species, including biotrophic, necrotrophic, and hemibiotrophic pathogens, were then tested for their ability to induce cell death using agroinfiltration in *N. benthamiana*. Of the 50 genes we cloned from pathogens, 18 triggered visible cell death. At least one protein each from the biotrophic pathogens *Sporisorium reilianum*, *Ustilago maydis*, and *Claviceps purpurea*, necrotrophic pathogens *Botrytis cinerea*, *Valsa mali*, *Verticillium dahliae*, and *Aspergillus flavus*, as well as hemibiotrophic pathogens *Sclerotinia sclerotiorum*, *Fusarium solani*, *Fusarium oxysporum*, and *Fusarium verticillioides* all induced cell death in *N. benthamiana* (Fig. 2j and Supplementary Data 2, 4). In addition, two proteins from fungi not associated with plants could also induce cell death in *N. benthamiana*. Based on these results, we concluded that Ser-Thr-rich GPI-anchored family proteins from diverse pathogens were recognizable by *N. benthamiana*.

### Cell death triggered by SGP1 in *N. benthamiana* requires BAK1

**Fig. 3:**
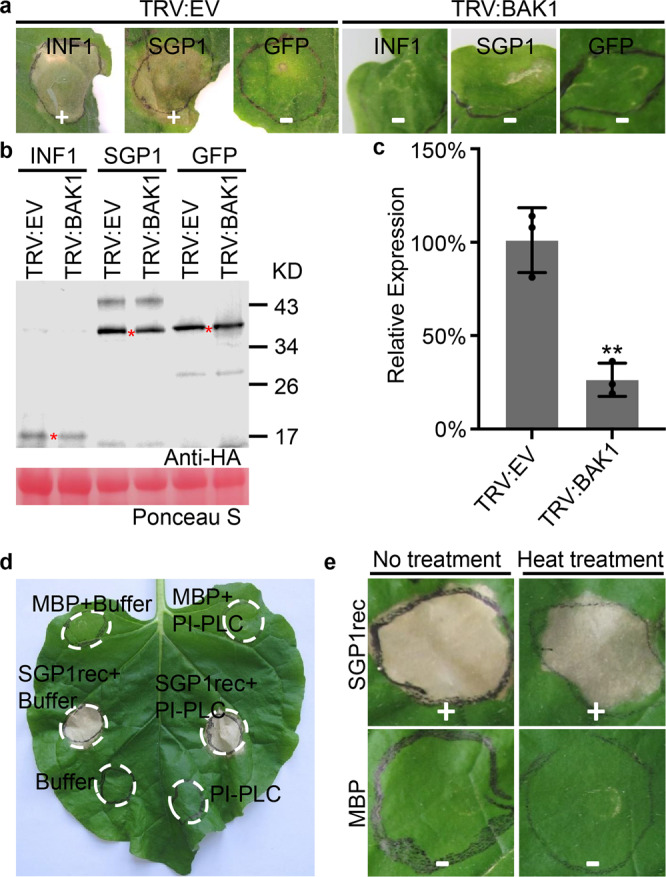
Cell death triggered by SGP1 requires BAK1 but is not affected after treatment with PI-PLC or heat in *N. benthamiana*. **a**–**c** Cell death triggered by SGP1 requires BAK1. **a** SGP1-triggered cell death in *TRV-silenced N. benthamiana*. Representative photographs were taken at 5 days post-infiltration. Data were obtained from 3 independent experiments, each of which used 10 VIGS-silenced plants per line (Supplementary Data 3). The plus symbol (+) indicates a positive cell death phenotype; minus symbol (−) indicates the absence of a clear cell death phenotype. **b** Western blot analysis of proteins transiently expressed in VIGS-silenced *N. benthamiana* leaves. The red asterisks (*) indicate the predicted protein sizes. **c** qRT-PCR quantification of relative transcript levels of *BAK1* in VIGS-silenced *N. benthamiana* leaves, normalized to those in TRV:*EV* lines, using the *EF1α* gene as an internal reference. Means and standard deviations obtained from three biological replicates. (**P < 0.01, two-sided *t*-test). **d** Cell death activity of SGP1rec in the presence or absence of PI-PLC, with MBP as a control. Data represent the results of three independent experiments in each of which one leaf was sampled and infiltrated from each of three plants per treatment (Supplementary Data 3). **e** Cell death response induced by heat-treated SGP1rec in *N. benthamiana* leaves. SGP1rec was treated under 100 °C for 10 min. Three independent experiments were performed (Supplementary Data 3), in each of which ten leaves were sampled per treatment. The plus symbol (+) indicates a positive cell death phenotype; minus symbol (−) indicates the absence of a clear cell death phenotype.

To determine whether BAK1, a central regulator of innate immunity, participates in the induction of cell death by SGP1, virus-induced gene silencing (VIGS) constructs were used to silence BAK1. The results showed that both INF1 and SGP1-triggered cell death in TRV:*EV* lines (Fig. 3a and Supplementary Data 3), as anticipated, while both failed to trigger cell death in *BAK1*-silenced plants. Western blot confirmed the successful expression of SGP1 in *TRV* transgenic lines (Fig. 3b). Quantitative real-time PCR (qRT-PCR) relative expression analysis revealed that *BAK1* transcripts were markedly reduced in TRV:*BAK1* lines compared with TRV:*EV* lines (Fig. 3c). These results suggested that *BAK1* was involved in SGP1-triggered cell death in *N. benthamiana*.

### SGP1-triggered cell death is not affected by phospholipase C treatment or heat treatment

SGP1 was predicted to have the essential characteristics necessary for GPI modification. To determine whether SGP1 was modified by GPI, we performed phase partition assays to test whether SGP1 protein moved from the hydrophobic detergent phase to the aqueous phase following phosphatidylinositol-specific phospholipase C (PI-PLC) treatment. Several forms of SGP1, including potential glycosylated- or polymers of SGP1, were detected in the aqueous phase after treatment with PI-PLC, whereas little protein was detected in the aqueous phase in the absence of PI-PLC (Supplementary Fig. 3a). An ~80 kDa band was present in the aqueous phase after PI-PLC treatment but not in the detergent phase (Supplementary Fig. 3a), which we hypothesized could be a previously unrecognized form of SGP1 protein released from the detergent phase. These results suggested that SGP1 was modified by GPI when expressed in *N. benthamiana*. To test whether the GPI modification was also involved in the induction of cell death by SGP1, we next infiltrated PI-PLC into *N. benthamiana* leaves 24 h after *Agro*-infiltration (Supplementary Fig. 3b) or co-infiltrated PI-PLC together with SGP1rec into *N. benthamiana* leaves (Fig. 3d and Supplementary Data 3). The results indicated that SGP1-triggered cell death was not affected by PI-PLC treatment. In addition, SGP1rec was still able to trigger cell death in *N. benthamiana* leaves even after heat treatment by boiling for 10 min (Fig. 3e and Supplementary Data 3). Taken together, these results demonstrated that SGP1 could function as a thermostable protein elicitor of cell death in *N. benthamiana*.

### The N terminus of SGP1 contains the elicitor-active epitope

**Fig. 4:**
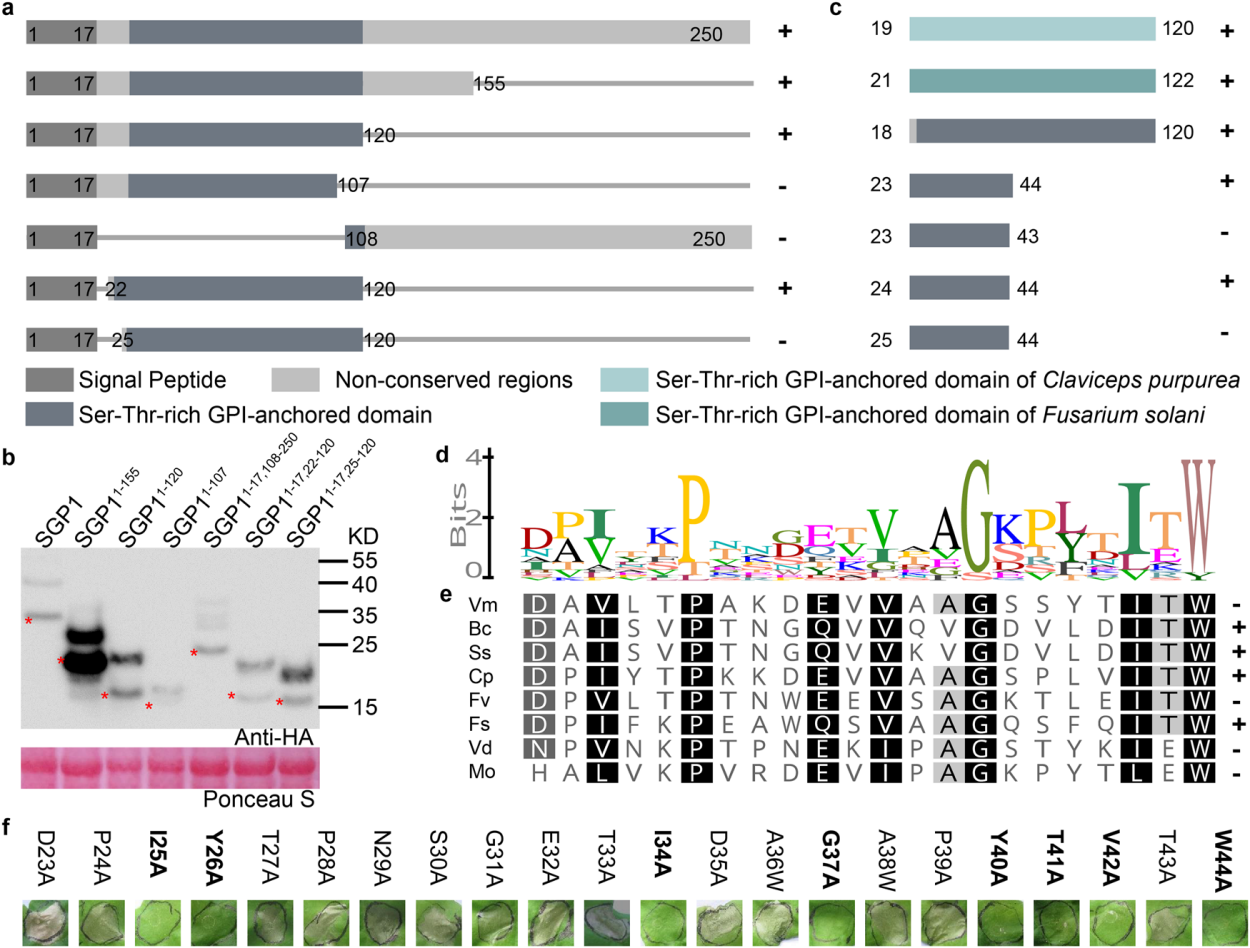
A 22 amino acid fragment in the N terminus of SGP1 is sufficient to mediate cell death in *N. benthamiana*. **a**–**c** Regions of SGP1 examined for cell death activity. Plus symbol (+), cell death phenotype; minus symbol (−), no clear cell death phenotype. Cell death phenotypes were recorded at 5 dpi. **a** Regions of SGP1 were examined for cell death activity by agrobacterium-mediated transient expression. Similar results were obtained from four independent experiments, with 10 plants per strain in each experiment (Supplementary Data 3). **b** Western blot analysis of SGP1 and truncated mutant variants transiently expressed in *N. benthamiana* leaves. The red asterisk (*) indicates the predicted protein sizes. **c** Regions of SGP1 examined for cell death activity in *N. benthamiana* by infiltration with 1 μM purified SGP1 variants from *E. coli*. Similar results were obtained from three independent experiments (Supplementary Data 3). **d** Sequence logos indicating the level of conservation for each residue of SNP22 region in GPI-anchored proteins that trigger cell death from plant-associated fungi. Sequence logos were constructed with Geneious R11 (Supplementary Data 6). The character and size of each logo represent the proportion of homologs with the amino acid at that specific site. **e**, **f** Cell death triggered by expression of recombinant SNP22 homolog proteins from other fungi and alanine-substituted versions of SNP22rec. **e** Sequence alignment and cell death-inducing activity of the SNP22 region of SGP1 homologs that can induce cell death. The homologous sequences of SNP22 from other fungi, including four amino acids flanking either terminus (Supplementary Data 7), were expressed in *E. coli*. *Vm*, KUI59516 (*Valsa mali*); *Bc*, Bcin01p05310.2 (*Botrytis cinerea*); *Ss*, EDO01950 (*Sclerotinia sclerotiorum*); *Cp*, CCE31100 (*Claviceps purpurea*); *Fv*, FVEG_00876T0 (*Fusarium verticillioides*); *Fs*, NechaP98847 (*Fusarium solani*); *Vd*, VDAG_JR2_Chr3g03370a-00001 (*Verticillium dahliae*); *Mo*, MGG_04599T0 (*Magnaporthe oryzae*). Three independent experiments were performed, using 10 plants for each SNP22 homolog (Supplementary Data 3) in each experiment. **f** Representative photos of cell death triggered by SNP22rec variants with single alanine substitutions at the indicated residues. Similar results were obtained from three independent experiments, with five plants per protein in each experiment (Supplementary Data 3).

To identify the location of the SGP1 protein epitope responsible for elicitor activity, we generated truncated mutant variants of SGP1 which carried an intact signal peptide and tested them for induction of cell death by agroinfiltration in *N. benthamiana*. As shown in Fig. 4a and Supplementary Data 3, the region containing residues 22–120, corresponding to the Ser-Thr-rich GPI-anchored domain of SGP1, fully triggered cell death in *N. benthamiana* leaves. Western blot showed that the bands corresponding to the predicted molecular weights were detectable in all of the truncated SGP1 variants (Fig. 4b). These results indicated that the elicitor-active epitope likely resides in the N terminus of SGP1. In addition, heterologous expression of the Ser-Thr-rich GPI-anchored domains of SGP1 homologs from *U. virens*, *C. purpurea*, or *F. solani* also induced cell death in *N. benthamiana* (Fig. 4c and Supplementary Data 3), thus indicating the participation of this conserved domain in triggering cell death. Moreover, the SGP1 N-terminus protein (SNPrec) was found to retain its ability to induce cell death even after heat treatment (Supplementary Fig. 4). Furthermore, sequence alignment of the full length of Ser-Thr-rich GPI-anchored proteins showed that the N-terminal sequences are more conserved than the C terminus, indicating a potentially conserved epitope located in the SGP1 N terminus (Supplementary Fig. 5 and Supplementary Data 4). Western blots (Fig. 4b) showed that the protein abundance of SGP1^1–107^ was lower than that of SGP1^1–120^, suggesting that the low abundance of SGP1^1–107^ may be related to its failure to induce cell death in *N. benthamiana*.

### A 22 amino acid peptide (SNP22) occurring in diverse fungal species is sufficient to induce ROS production and cell death in *N. benthamiana*

**Fig. 5:**
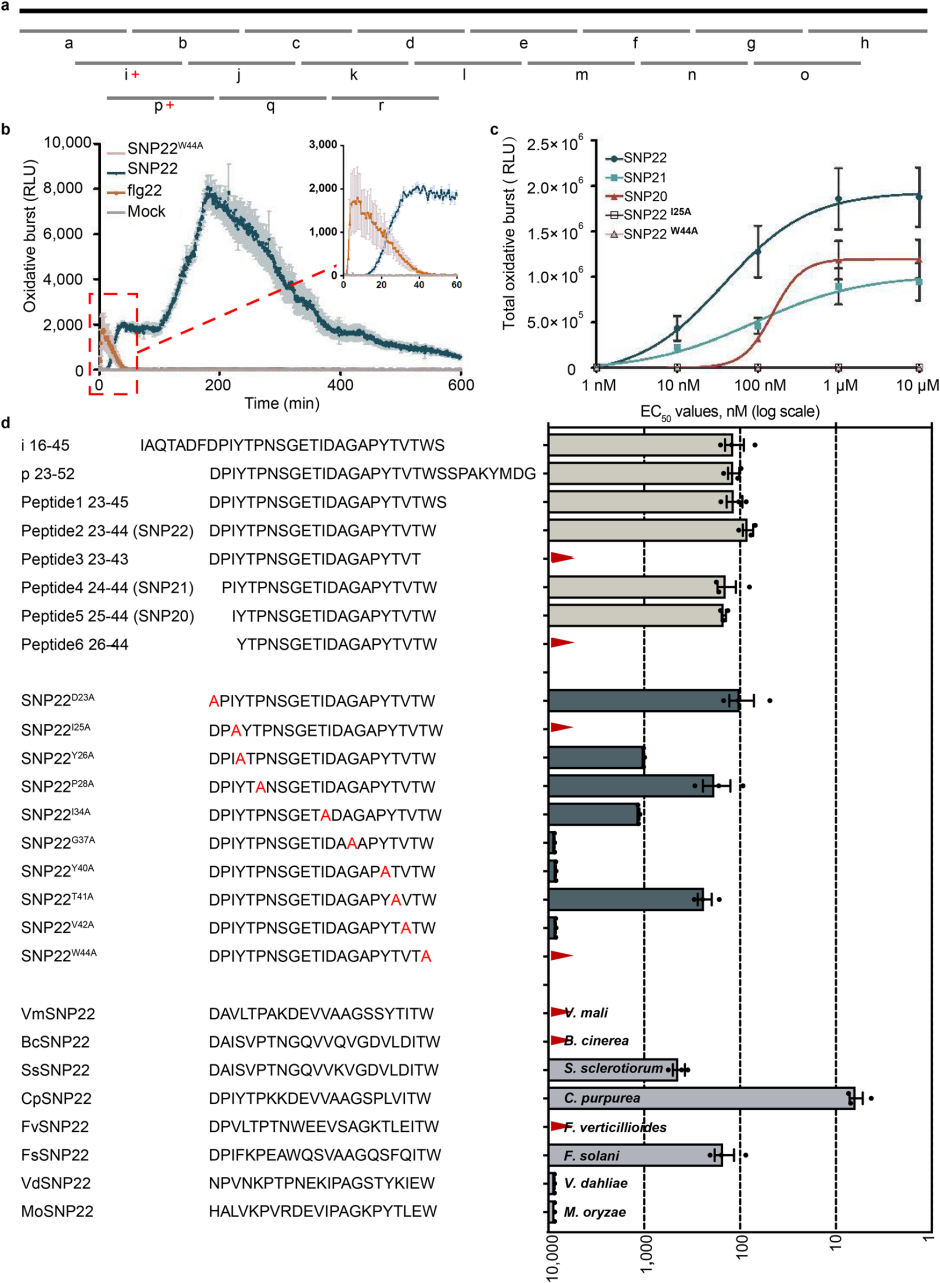
A 22 amino acid peptide (SNP22) of SGP1 is sufficient to induce ROS production in *N. benthamiana*. **a** Schematic representation of synthetic peptides (30-mer peptides, a–r) covering wild-type SGP1. **b** ROS production dynamics induced by 1 μM peptides of SNP22 and SNP22^W44A^ in *N. benthamiana* leaf discs. 100 nM flg22 and buffer served as positive and negative controls, respectively. ROS production was measured with a luminol-based assay. Leaf discs from 3- to 4-week-old *N. benthamiana* plants were incubated with the indicated peptides, and the luminescence was expressed as relative light units (RLU). Values represent means ± SEM of three leaf disks, each sampled from a different plant and tested in parallel. **c** Dose–response curves of total ROS production induced by SNP22, SNP21, SNP20, SNP22^I25A^, or SNP22^W44A^ peptides in *N. benthamiana* leaf discs. Values represent the means ± SEM of three independent experiments, each of which used three leaf disks per treatment. **d** Summary of EC_50_ values determined from dose–response curves based on total ROS production. Peptide names and sequences are indicated on the left. EC_50_ values of each peptide are shown on a logarithmic scale on the right. Values represent the means ± SEM of three independent experiments, with three leaf disks per treatment used in each experiment. No activity could be detected with peptides denoted by red triangles at 10 μM. *Vm*, KUI59516 (*Valsa mali*); *Bc*, Bcin01p05310.2 (*Botrytis cinerea*); *Ss*, EDO01950 (*Sclerotinia sclerotiorum*); *Cp*, CCE31100 (*Claviceps purpurea*); *Fv*, FVEG_00876T0 (*Fusarium verticillioides*); *Fs*, NechaP98847 (*Fusarium solani*); *Vd*, VDAG_JR2_Chr3g03370a-00001 (*Verticillium dahliae*); *Mo*, MGG_04599T0 (*Magnaporthe oryzae*).

To further identify the minimal immunogenic epitope within SGP1, nested peptides covering the entire SGP1 protein sequence were synthesized and tested for their abilities to trigger reactive oxygen species (ROS) production in *N. benthamiana* leaf disks (Fig. 5a). Two overlapping peptides of SGP1, i and p, spanning residues I16 to S45 and D23 to G53, respectively, clearly showed the ability to induce an ROS burst. These peptides overlapped from D23 to S45 (DPIYTPNSGETIDAGAPYTVTWS, peptide 1, Fig. 5d), suggesting that this fragment constitutes the core of the immunogenic activity of SGP1. Synthetic peptides progressively shortened by C-terminal or N-terminal deletions were further analyzed to determine the minimal length required for immunogenic activity (Fig. 5b–d). The EC_50_ value determined for SNP22 was similar to that of peptide 1, while the EC_50_ values of SNP21 and SNP20 were nearly twice that of SNP22. Peptide 3, which lacks W44, and peptide 6, which lacks I25, did not induce ROS production even at 10 μM concentrations (Fig. 5d). The total ROS production triggered by SNP22 was also nearly twofold greater than that of SNP21 and SNP20 (Fig. 5c). These results indicated that the 22 amino acid peptide was sufficient to induce ROS production, and the minimal peptide capable of inducing an ROS burst in *N. benthamiana* could be as small as 20 amino acids.

To test whether SNP22-like peptides of GPI-anchored protein from other fungi harbor MAMP activity, we next analyzed the immunogenic potential of synthetic peptides that represented other fungi-derived sequences orthologous to SNP22 of UvSGP1. The results showed that FsSNP22, CpSNP22, and SsSNP22 all induced ROS production, while VmSNP22, BcSNP22, and FvSNP22 did not, even at 10 μM concentrations (Fig. 5d). The synthetic peptides were also tested for their cell death-inducing activity in *N. benthamiana* leaves and only CpSNP22 was found to induce an obvious cell death phenotype (Supplementary Fig. 6 and Supplementary Data 3). The inconsistency in the induction of ROS production and the induction of cell death between the synthetic peptides and their corresponding recombinant full-length proteins may be caused by differences in stability, folding, or conformation^15,17,41^. To address this issue, we then used recombinant SNP22 (SNP22rec) to test the ability of this peptide to trigger cell death and found that SNP22rec could indeed induce cell death in *N. benthamiana* (Fig. 4c). Furthermore, a minimal 21 amino acid region of this recombinant protein (SGP1^24-44^rec) was enough to induce cell death (Fig. 4c and Supplementary Data 3). Then, to investigate whether this 22 amino acid region in other fungi could also induce cell death, we tested homologous recombinant SNP22 proteins (plus 4 flanking residues at each terminus) from other plant-associated fungi. These experiments showed that BcSNP22rec, ScSNP22rec, FsSNP22rec, and CpSNP22rec could all induce cell death in *N. benthamiana* (Fig. 4d, e and Supplementary Data 3, 6, 7), indicating that the cell death-inducing activity of this region is conserved across SGP1 homologs.

To determine which amino acids were required for MAMP activity of the SNP22 region, we determined the cell death-inducing activity of SNP22rec variants carrying single alanine substitutions at each of the 22 residues (Fig. 4f and Supplementary Data 3). The results suggested that I25, Y26, I34, G37, Y40, T41, V42, and W44 residues are required for its induction of programmed cell death, among which I25, I34, G37, V42, and W44 were the most conserved residues (Fig. 4d, f and Supplementary Data 3, 6). In addition, analysis of ROS production triggered by synthetic SNP22 variants with single alanine substitutions at the conserved amino acids or at residues found to be necessary for inducing cell death, suggested that I25, Y26, I34, G37, Y40, V42, and W44 residues are required for SNP22 activation of ROS production (Fig. 5b–d). These results suggested that the I25, Y26, I34, G37, Y40, V42, and W44 residues may be required for receptor recognition of SGP1 to induce cell death and ROS production.

Next, to test whether the SNP22 fragment determines the full activity of SGP1 in inducing cell death and ROS production, we generated the recombinant SGP1 mutants SGP1^I25A^rec and SGP1^W44A^ rec and found that neither SGP1^I25A^rec nor SGP1^W44A^ rec was able to induce cell death or ROS production (Supplementary Fig. 7 and Supplementary Data 3). Thus, the alanine substitutions in I25 or W44, which abolished the elicitor ability of SNP22rec, also abolished the activity of the full-length SGP1rec, indicating that the SNP fragment is the determinant of SGP1 elicitor activity. Taken together, we can infer that SNP22 could serve as a minimal immunogenic epitope of SGP1 in MAMP activity.

### SNP22 triggered the induction of disease resistance in *N. benthamiana* and rice

**Fig. 6:**
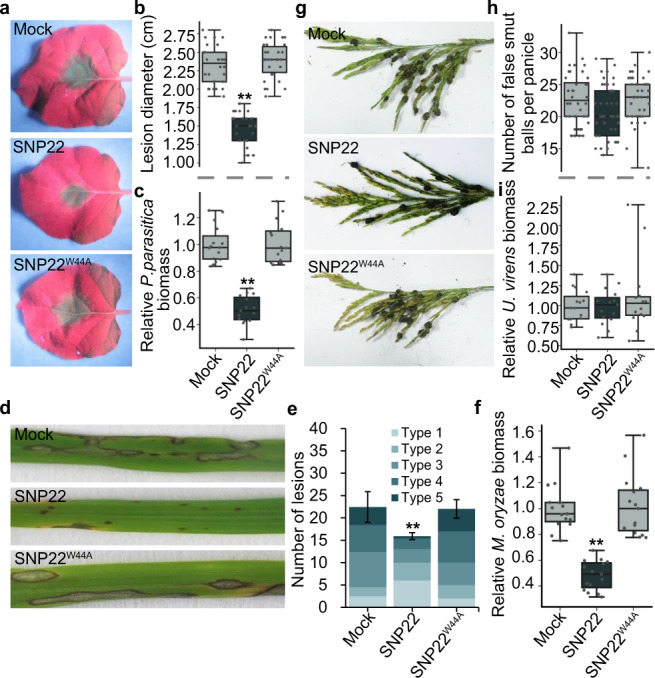
SNP22 triggers induction of disease resistance in *N. benthamiana* and rice. *N. benthamiana* leaves, rice panicles, and rice leaves were pretreated with 1 μM of the indicated peptide for 24 h before pathogen inoculation. Buffer served as a mock treatment. In box plots, whiskers indicate the minimum and maximum values, line indicates the median, the box boundaries indicate the upper (25th percentile) and lower (75th percentile) quartiles. (**P < 0.01 compared with Mock, two-sided Dunnett’s test). **a**–**c** Induction of disease resistance to *P. parasitica var nicotianae* in *N. benthamiana*. **a** Lesions were assessed under UV light and representative photographs were taken at 48 h post inoculation (hpi). **b** Statistical analysis of lesion diameters. Data was obtained from six independent experiments, each of which tested five leaves for each treatment. **c** Relative biomass of *P. parasitica* in *N. benthamiana* leaves quantified by qPCR analysis of genomic DNA and normalized to mock treatment. Values from five biological replicates were shown, which include three technical replicates for each biological replicate. **d**–**f** Induction of disease resistance to *M. oryzae* in rice leaves. **d** Representative photographs were taken at 7 dpi. **e** Quantification of lesion types per 5 cm leaf strip. See Methods for description of lesion types. Values represent the means ± SD of five independent experiments, each of which used 10 leaves for each treatment. **f** Relative biomass of *M. oryzae* in rice leaves, quantified by qPCR analysis of genomic DNA and normalized to mock treatment. Values from five biological replicates were shown, which include three technical replicates for each biological replicate. **g**–**i** Disease resistance to *U. virens* in rice panicles. **g** Representative photographs were taken at 30 dpi. **h** Quantification of false smut balls per panicle. The data were collected from five independent experiments, with a total of at least 44 panicles per line. **i** Relative biomass of *U. virens* in rice spikelets, measured by qPCR analysis of genomic DNA and normalized to mock treatment. Values from five biological replicates were shown, which include three technical replicates for each biological replicate.

To determine whether SNP22 triggers immunity in non-host *N. benthamiana*, we firstly determined whether SNP22 pretreatment could enhance the resistance of *N. benthamiana* to *Phytophthora parasitica* var *nicotianae*. We found that lesion diameter and relative *P. parasitica* biomass in *N. benthamiana* leaves were significantly reduced in plants pretreated with SNP22 peptide compared to mock and SNP22^W44A^ pretreatment (Fig. 6a–c). There were no detectable, direct effects of SNP22 on *P. parasitica* growth on plates, indicating that SNP22 peptides did not exert any direct toxicity against *P. parasitica* (Supplementary Fig. 8). We then investigated whether SNP22 could induce defense responses other than disease resistance. Compared with control and SNP22^W44A^ peptides, the relative expression of defense-related genes and ROS accumulation were enhanced after treatment with SNP22 peptide (Supplementary Fig. 9). Taken together, these results indicated that SNP22 triggered the activation of a canonical defense response signal cascade and disease resistance in *N. benthamiana*.

To further evaluate the biological significance of the SNP22 region in the cognate host, we determined if resistance was successfully induced against *Magnaporthe oryzae* in leaves and against *U. virens* in rice panicles. As shown in Fig. 6d–f, *M. oryzae* lesions and biomass on rice leaves were significantly reduced following SNP22 pretreatment compared to mock or SNP22^W44A^ pretreatment. Nevertheless, compared with mock or SNP22^W44A^ peptide pretreatment, no obvious differences could be detected in the number of false smut balls per panicle or in the relative *U. virens* biomass after SNP22 pretreatment (Fig. 6g–i). Taken together, these results indicated that SNP22 could trigger the induction of disease resistance in rice leaves to *M. oryzae* but not in rice panicles to *U. virens*.

### Responses to SNP22 differ between leaves and panicles of rice

**Fig. 7:**
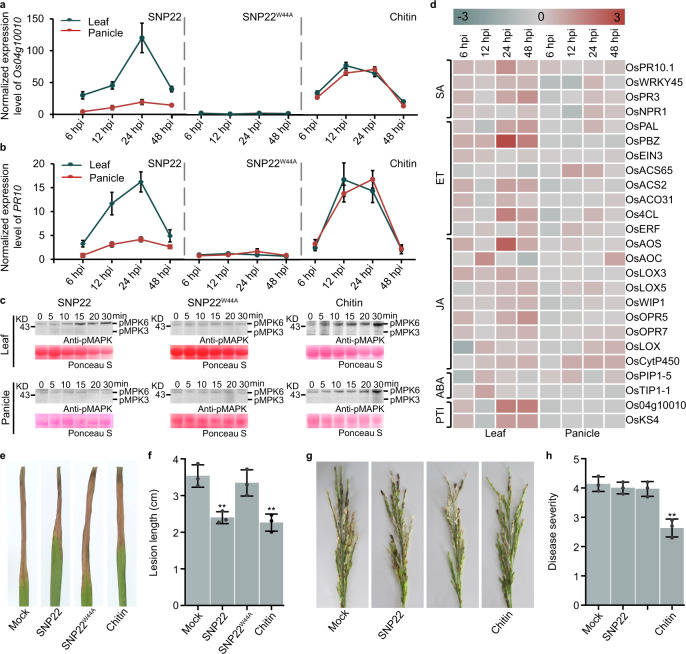
Immune responses triggered by SNP22 in rice leaves and panicles. **a**–**c** Rice leaves and panicles were treated with either 1 μM SNP22 peptide, SNP22^W44A^ peptide, or chitin (hexa-*N*-acetylchitohexaose). **a**, **b** Relative induction of defense-related gene expression in rice leaves and panicles triggered by the indicated elicitors. Defense-related gene expression was normalized to that of mock at each time points after treatment. Rice *actin* served as an internal reference. Values represent the means ± SEM of three biological replicates. **c** MAPK activation in rice leaves and panicles triggered by elicitors. The phosphorylation level of MAPK detected by immunoblot with anti-phospho-p44/42 MAPK (Erk1/2) antibody. **d** Transcriptome analysis. The relative gene expression was normalized to those of mock treatment at each time point post-treatment. Heatmap was shown based on lg values of the fold change. Cyan color, downregulation; red color, upregulation. **e**–**h** Disease resistance to *X. oryzae pv. oryzae* in rice leaves and to *B. glumae* in rice panicles triggered by the indicated elicitors. Rice leaves and panicles were pretreated with either 1 μM SNP22, SNP22^W44A^, or chitin 24 h before pathogen inoculation. Symptoms were assessed at 7 dpi. Three independent experiments were performed, and ten leaves or ten panicles were used for each experiment. **e** Representative photographs of symptoms on rice leaves caused by *X. oryzae pv. oryzae*. **f** Lesion lengths. Values represent the means ± SD of three independent experiments, with ten leaves in each experiment (**P < 0.01 compared with Mock, two-sided Dunnett’s test). **g** Representative photographs of disease symptoms on rice panicles caused by *B. glumae*. **h** Disease severity in rice panicles, evaluated as described in the “Methods” section. Values represent the means ± SD of three independent experiments, with 10 panicles per treatment in each experiment (**P < 0.01 compared with Mock, two-sided Dunnett’s test).

To assess whether SNP22-induced plant defense differs between leaves and panicles, we examined the activation of the defense-related gene, mitogen‐activated protein kinase (MAPK), and ROS production in rice leaves and panicles. As shown in Fig. 7a, b and Supplementary Fig. 10a, the activation of pattern-triggered immunity (PTI) marker gene Os04g10010^42^, salicylic acid (SA) responsive gene *PR10*, together with ethylene (ET) responsive gene *phenylalanine ammonia lyase* (*PAL*), was stronger in leaves than in panicles after treatment with SNP22 peptide, compared with that in SNP22^W44A^-treated plants. MAPK phosphorylation levels (Fig. 7c) and ROS production (Supplementary Fig. 10b) increased significantly in rice leaves but were not significantly changed in rice panicles after treatment with SNP22 peptide. To further assess the differences in immunity induced by SNP22, we next performed RNA-seq. As shown in Fig. 7d, overall, PTI marker, SA-, ET-, jasmonic acid (JA)-, and abscisic acid (ABA)-related defense genes were induced after SNP22 treatment. In addition, the fold changes in the upregulation of most defense-related genes were substantially higher in leaves than in panicles, although with some exceptions such as *NPR1, OsACS65, OsERF, OsLOX*, and *OsCytP450*. Similar transcriptional patterns of *Os*04g10010, *PR10,* and *PAL* were observed in qRT-PCR assays, thus validating the RNA-seq results (Fig. 7a, b and Supplementary Fig. 10a). Taken together, we infer that SNP22 induced stronger responses in rice leaves than in panicles.

To further confirm the differences in SNP22-triggered resistance between rice leaves and panicles, we also tested SNP22-mediated resistance to rice pathogens *Xanthomonas oryzae pv. oryzae (Xoo)* and *Burkholderia glumae*, which causes bacterial leaf blight and bacterial panicle blight in rice, respectively. As shown in Fig. 7e–h, pretreatment with SNP22 led to reduced disease symptoms in leaves caused by *Xoo*, but not in panicle symptoms caused by *B. glumae*. Pretreatment with SNP22^W44A^ showed no impact on disease symptoms caused by either pathogen. These results suggested that SNP22 appeared to induce an effect in which rice leaves exhibited resistance to pathogen infection while panicles did not.

To examine whether the unequal immune response between rice leaves and panicles triggered by SNP22 pretreatment is a general phenomenon, we examined the immune responses triggered by chitin in both organs. We observed that defense-related genes, MAPK, and ROS production were activated in both leaves and panicles after treatment with chitin, but these markers were not enhanced in either leaves or panicles after pretreatment with SNP22^W44A^ (Fig. 7a–c and Supplementary Fig. 10). In addition, pretreatment with chitin resulted in a comparable reduction in disease symptoms in both leaves (caused by *Xoo)* and panicles (caused by *B. glumae*) (Fig. 7e–h). Collectively, these results suggested that the unequal immune response we observed between rice leaves and panicles triggered by SNP22 pretreatment does not appear to be a general phenomenon associated with exposure to different MAMPs.

### SGP1 is required for *U. virens* pathogenicity during rice panicle infection, but it appears to induce immunity during *U. virens* challenge of rice leaves

**Fig. 8:**
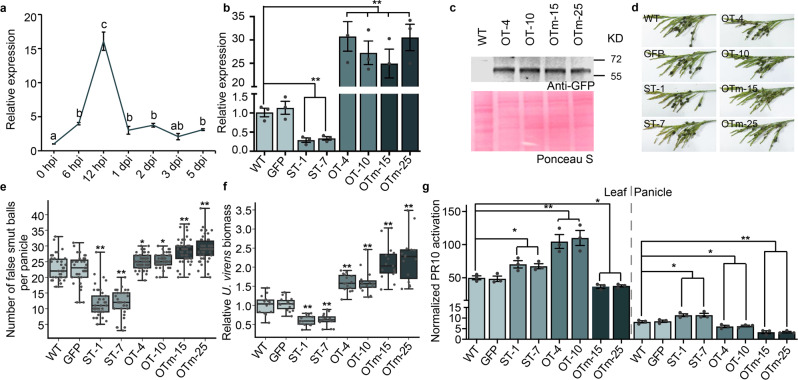
Involvement in pathogenicity and elicitor function of SGP1 during *U. virens* infection of rice panicles and challenge of rice leaves. **a** The relative expression of *SGP1* during different stages of infection. *SGP1* expression was normalized to its expression level prior to inoculation (0 hpi) using *Tubulin* as an internal reference. hpi, hours post inoculation; dpi, days post inoculation. Values are the means ± SEM of three biological replicates. The different letters indicate statistically significant differences (*P* < 0.05) as measured by Duncan’s multiple range test. **b** Relative transcript levels of *SGP1* in different *U. virens* transgenic lines. The transcript levels were measured by qRT-PCR and normalized to those in the WT strain using *Tubulin* as an internal reference. ST, *SGP1-*silenced transformants of *U. virens*; OT, *SGP1* overexpression transformants of *U. virens*; OTm, *SGP1*^*W44A*^ overexpression transformants of *U. virens*. Values are the means ± SEM of three biological replicates. (**P < 0.01; two-sided *t*-test). **c** Western blot analysis of SGP1 and SGP1^W44A^ in *U. virens* WT and transgenic lines with anti-GFP antibody. **d**–**f** Pathogenicity phenotypes of transgenic *U. virens* lines and WT. In box plots, whiskers indicate minimum and maximum values, line indicates the median, the box boundaries indicate the upper (25th percentile) and lower (75th percentile) quartiles. **d** Representative photographs of disease symptoms on rice panicles. **e** Quantification of the number of false smut balls per panicle. Data were collected from five independent experiments, with a total of at least 31 panicles per line. (*P < 0.05; **P < 0.01 compared with WT, two-sided Dunnett’s test). **f** Relative *U. virens* biomass in infected spikelets, measured by qPCR of genomic DNA and normalized to WT. Values from five biological replicates were shown, which include three technical replicates for each biological replicate. (**P < 0.01 compared with WT, two-sided Dunnett’s test). **g** Relative induction of *PR10* transcription in rice leaves and panicles at 12 hpi. The induction of PR10 expression in *U. virens*-inoculated rice was normalized to that in mock-inoculated rice, with *Actin* serving as an internal reference. Values are the means ± SEM of three biological replicates. (**P < 0.01; *P < 0.05; two-sided *t*-test).

To determine the biological role of SGP1 during *U. virens* infection, we analyzed the expression patterns of *SGP1* during different infection stages. The upregulation of *SGP1* during the early infection stages (Fig. 8a) suggested the potential involvement of SGP1 in *U. virens* infection. Next, we obtained *SGP1-*silenced transformants (ST-1 and ST-7), and *SGP1* overexpression (OT-4 and OT-10) or SGP1^W44A^ overexpression (OTm-15 and OTm-25 -with abolished SGP1-associated MAMP activity) transformants in *U. virens*. *U. virens* lines expressing GFP served as transgenic controls. The relative transcript and protein levels of SGP1 in *U. virens* transformants were confirmed by qRT-PCR or western blot, respectively (Fig. 8b, c). All transformants showed normal filamentous growth, conidial morphology, and hyphal morphology compared with WT or the GFP transgenic control (Supplementary Fig. 11). However, compared to WT and GFP strains, the number of false smut balls per panicle and the relative pathogen biomass in the two SGP1-silenced transformants were significantly reduced, while those caused by transformants overexpressing SGP1 or SGP1^W44A^ were increased (Fig. 8d–f). These results indicated that SGP1 is required for pathogenicity of *U. virens* during infection of rice panicles.

To determine whether SGP1 is recognized by rice during *U. virens* challenge, activation of *PR10* gene expression was determined in both leaves and panicles at 12 hpi with *U. virens* transgenic lines or controls. The results showed that the *U. virens* WT strain could induce clearly unequal responses in leaves versus panicles of rice, with an approximately 50-fold increase in PR10 expression in rice leaf tissues but only about 10-fold increase in panicles (Fig. 8g). Compared to the WT and GFP transgenic line, the activation of PR10 in leaves was significantly increased in response to OT-4 and OT-10 lines but decreased in response to OTm-15 and OTm-25 lines. These results indicated that SGP1 may have a role in activating *PR10* in rice leaves during *U. virens* challenge. Unlike the triggering of PR10 activation in response to OT-4 and OT-10 lines compared to WT strain in rice leaves, *U. virens*-induced PR10 activation was lower in response to OT-4 and OT-10 lines compared to WT and GFP transgenic lines in panicles (Fig. 8g). These results indicated that SGP1 does not appear to induce the activation of PR10 in panicles during *U. virens* infection. Compared to WT and GFP transgenic lines, the activation of PR10 in panicles is significantly increased in response to ST-1 and ST-7 lines but decreased in response to OTm-15 and OTm-25 lines, similar to the response in leaves. These results suggested that SGP1 may also potentially contribute to suppressing PR10 activation during infection. Taken together, these results suggested that SGP1 appears to activate an immune response in rice leaves, whereas it contributes to *U. virens’* virulence during infection of rice panicles.

## Discussion

Some pathogens infect only a specific organ or set of organs, and this organ-specific innate immunity has been well-documented in mammals. Plant pathogens are also reported to infect their hosts through specific organs, such as *U. virens*, suggesting that different organs may exhibit variation in their inherent capacity to resist invasion. Here, we identified the thermostable proteinaceous MAMP, SGP1, a Ser-Thr-rich GPI-anchored family protein that is widely distributed across fungal taxa. Moreover, a minimal peptide of 22 amino acids in the N terminus constitutes the immunogenic epitope of SGP1, the detection of which by a rice plant host results in an unequal, and distinctly stronger response by leaves than panicles. Notably, we found that during *U. virens* infection of rice *in planta*, SGP1 is required for *U. virens* infection of rice panicles, but appears to activate immunity in rice leaves.

GPI-anchored proteins are structurally and functionally diverse and play vital roles in numerous biological processes^28,29^. In mammals, prion protein (PrP), a widely expressed and conserved GPI-anchored protein, causes a neurodegenerative disease when misfolded variants accumulate in cells^43–45^. In plants, GPI-anchored proteins are required for both development and immunity signaling. For example, LORELEI (LRE), which encodes a putative GPI-anchored surface protein, together with FERONIA, jointly function in pollen tube reception^46^. In the human pathogen *Candida albicans*, the GPI-anchored protein CaEcm33p is required for cell wall integrity, morphogenesis, and virulence^47^. The function of GPI-anchored proteins in plant pathogenic fungi has only recently been reported. The *Aspergillus flavus* GPI-anchored protein encoded by *ecm33* participates in growth, development, aflatoxin biosynthesis, and maize infection^48^. In our study, we showed that SGP1 contrastingly functions as a recognizable MAMP, triggering innate immunity in rice leaves, but is required for *U. virens* pathogenicity during infection of panicles.

The amino acids in SNP22rec required for triggering cell death are also required for full activation of the ROS burst by SNP22 peptide (Figs. 4 and 5). It is possible that SNP22 is recognized by one receptor that initiates both signaling outcomes. The minimal peptide for triggering cell death in *N. benthamiana* is 21 amino acids (P24-W44), while the minimal peptide for triggering ROS production is 20 amino acids (I25-W44). Deletion of the D23 and P24 residues from SNP22, to create SNP20, resulted in impaired ability to induce cell death, whereas single alanine substitutions at either residue did not attenuate SNP22 cell death-inducing activity, indicating that neither D23 nor P24 are likely receptor binding sites. It is also possible that the attenuation of SNP20-activated ROS production compared to SNP22 may be a function of its lower stability in the apoplast. Since SNP20 leads to ROS production but not cell death in *N. benthamiana*, it is possible that D23 to P24 may serve as structural components that maintain a MAMP configuration that triggers cell death. In addition, cell death triggered by SNP22 raises the issue that induction of cell death is not restricted to ETI signaling but can also occur as part of a PTI response. Typically, ETI is associated with cell death and systemic acquired resistance, while PTI is not. However, SGP1, together with the MAMPs CBEL, EIX, XEG1, and harpins all induce cell death in plants^23,49–52^, indicating that programmed cell death is not exclusive to ETI, and that the distinction between PTI and ETI signaling may be blurry.

Organ-specific regulation of innate immunity has been described in mammals and involves complex processes with multiple contributing factors that potentially control the responsiveness of tissues or organs to MAMPs. Organ-specific responses may be dependent on differences in the threshold for receptor activation related to differences in the organ- or tissue-specific expression profiles of pattern recognition receptors (PRR), which enables different degrees of cellular-level regulation of their respective tissue microenvironments. In addition, the expression of negative regulators of TLRs is reported to vary between different organs, and ultimately these microenvironment factors converge so that TLR signaling is modulated in an organ-specific manner^53,54^. Many phytopathogenic fungi are commonly reported to infect their hosts through specific organs^55^. Organ- or tissue-specific immunity has been reported in plants, although the mechanisms are far from clear. Dirk *et al*. reported organ-specific defense responses induced by *Colletotrichum graminicola* in maize, including production of antimicrobial flavonoids and differential expression of defense- and stress-related genes^56^. *Arabidopsis* root tissues have the capacity to activate immune responses triggered by flg22^57,58^. However, significant differences have been found in the intensity of these responses and in the expression of the FLS2 receptor across various root tissues, potentially due to the density of receptor distribution^59^. In our study, we observed a phenomenon in which SNP22 appears to induce a stronger response in leaves compared to panicles. In vivo experiments with transgenic *U. virens* lines demonstrated that SGP1 induces immunity in rice leaves but not in rice panicles during pathogen challenge of rice. By contrast, chitin triggered comparable defense responses in both rice leaves and panicles, indicating that the differences in SGP1 MAMP activity in rice are possibly organ-specific. However, we cannot rule out that any differences in penetration between MAMPs might contribute to the differences in the generality of the response. Further, we provide the caveat that potential differences in penetration by exogenously applied SNP22 could contribute to the differences in response between leaves and panicles, and that exogenous application may not perfectly replicate the distribution of the elicitor during infection.

It warrants consideration that the stronger immune response in rice leaves compared to panicles triggered by this MAMP may potentially correlate with the adaptation of *U. virens* to panicle instead of leaf infection. Differences in MAMP-triggered immunity between organs may thus be one possible contributing factor to organ-specific specialization by pathogens. It is also possible that some pathogens have evolved organ-specific virulence factors that drive their specialized pattern of infection, thus organ-specific virulence may be an alternative explanation for organ-specific differences in infection by some pathogens. However, SGP1 is widely distributed in fungi, and among those plant pathogens that carry it, not all use an organ-specific manner of infection, which thus suggests that pathogens may evolve different strategies to escape host immune recognition or modulate host immunity. Further investigation of the molecular mechanisms of receptor recognition of SGP1 as a MAMP, as well as modulation of plant immunity by SGP1 as a virulence factor, will improve our understanding of specialized pathogens with organ-specific manners of infection.

## Methods

### LC–MS/MS analysis of secreted proteins from *U. virens*

LC–MS/MS analysis was performed by Beijing Bangfei Bioscience & Technology Co., Ltd. Specifically, 10-day-old *U. virens* culture supernatants were collected and filtered through a 0.22-µm polyvinylidene fluoride membrane (Millipore). Proteins were precipitated overnight at 4 °C by adding 20% TCA. Then the samples were centrifuged for 15 min at 15,000 × *g* at 4 °C. Cold acetone was added to the resultant pellets, and proteins were precipitated for 30 min at 4 °C, followed by centrifugation as above. The wash step was repeated once. The protein precipitates were air-dried for 1–3 min and dissolved in a buffer containing 8 M Urea, 100 mM Tris-HCl (pH 8.0), and 1× protease inhibitor cocktail (Roche) by incubating at 4 °C overnight. Proteins were harvested by centrifuging at 10,000×*g* at 4 °C for 3 min. The proteins were digested by trypsin (Promega) followed by analysis with the Orbitrap Fusion Lumos mass spectrometer (ThermoFisher Scientific, Waltham, MA, USA) coupled with a nano high-performance liquid chromatography system (UltiMate 3000 LC Dionex; ThermoFisher Scientific). The peptides were loaded onto a C18-reversed-phase column (3 μm C18 resin, 100 μm × 20 mm) and separated on an analytical column (1.9 μm C18 resin, 150 μm × 120 mm; Thermo Fisher Scientific) using mobile phase A: 0.5% formic acid /H_2_O and B: 0.5% formic acid/acetonitrile at a flow rate of 300 nL/min, using a 150 min gradient. Peptide identification was performed with the SEQUEST search engine of Proteome Discoverer 2.1 (Thermo Fisher Scientific) software using uniport-*U. virens* sequences downloaded from Uniprot (UP000027002) with the false discovery rate for peptides and proteins set to 0.01. The proteins and the peptides that met the successively stringent criteria mentioned in the results are shown in Supplementary Data 1.

### Bioinformatics

Signal peptides were identified by the SignalP 3.0 server (http://www.cbs.dtu.dk/services/SignalP-3.0/). Protein domains were predicted by Pfam (https://pfam.xfam.org/). For the phylogenetic tree of fungal species, the orthologous proteins between species were predicted by OrthoFinder v 2.2.6^60^ with default settings. Pairwise sequence similarities were calculated by performing all-against-all BLASTP comparisons with an *E*-value cutoff of 1e−5. Orthologous groups that contained only one gene for each species were selected to construct the phylogenetic tree of fungal species (Supplementary Data 5). Multiple sequence alignments were performed using the ClustalW2 program with default parameters. Maximum-likelihood phylogenetic dendrograms were constructed using PhyML v3.0^61^ and graphically viewed using MEGA 6 (www.megasoftware.net)^62^. The phylogenetic tree of proteins that contain the GPI-anchored domain (Supplementary Data 2) was constructed with Geneious R11 using the neighbor-joining method. Sequences of different fungi, bacteria, oomycetes, and plants were downloaded from Ensembl Genomes (https://ensemblgenomes.org). Cartoons were downloaded from the website https://www.flaticon.com or https://icons8.com/icons. GPI modification was predicted by the programs PSORT (https://wolfpsort.hgc.jp/), PredGPI (http://gpcr.biocomp.unibo.it/predgpi/pred.htm), and big-PI Plant Predictor (https://mendel.imp.ac.at/gpi/cgi-bin/gpi_pred_plants.cgi).

### Plasmid construction and preparation

A mixture of cDNAs from *U. virens* collected at different infection stages was used as a PCR template to amplify the coding sequences of *U. virens* genes using Phanta Super-Fidelity DNA Polymerase (P501-d1, Vazyme). Genes were cloned into a vector based on homologous recombination technology using the Vazyme ClonExpress II One Step Cloning Kit (C112, Vazyme). The primers and constructs used in this study are listed in Supplementary Data 8 and 9, respectively. For *Agrobacterium*-mediated transient expression in *N. benthamiana*, the coding sequences of *U. virens* secreted proteins were separately cloned into the binary PVX vector pGR107, which adds a C-terminal 3*HA tag to the proteins. pGR107:GFP and pGR107:INF1 plasmid constructs served as negative and positive controls, respectively. For expression of the protein in *E. coli*, mature sequences encoding SGP1 or the mutant forms of SGP1 were cloned to the pHMTc vector, which contains an N-terminal His tag and MBP protein. For expression of the protein in *U. virens*, sequences encoding SGP1 or SGP1 mutants were cloned into the pKD1-GFP vector, which contains a C-terminal GFP tag.

### *Agrobacterium* and protein/peptide infiltration assays

*Agrobacterium*-mediated transient expression assays were performed to test the cell death-inducing activity of proteins. Genes cloned into the pGR107 vector were transformed into *Agrobacterium* cells and then infiltrated into *N. benthamiana* leaves using a needleless syringe at a concentration of 0.5 OD. *Agrobacterium* strains carrying GFP and INF1 pGR107 constructs were infiltrated in parallel as negative and positive controls, respectively. For protein infiltration assays, the protein was expressed and purified from *E. coli* strain Rosetta using Ni-NTA Superflow resin (Qiagen) following the manufacturer’s instructions. Protein was first adjusted to the appropriate concentration and then infiltrated into plant leaves of different species using a needless syringe. SGP1 protein solutions ranging from 1.5 nM to 1.5 μM were infiltrated into the leaves of *N. benthamiana*. MBP protein expressed from the empty vector served as a negative control. Protein solutions (1 μM) were also infiltrated into the leaves of different plants. Photos were taken at 2 dpi for potato plants (*Solanum tuberosum*) and at 7 dpi for tomato (*Solanum lycopersicum*), pepper (*Capsicum annum*), maize (*Zea mays*), wheat (*Triticum aestivum*), soybean (*Glycine max*), and cotton (*Gossypium hirsutum*) plants. For the peptide infiltration assays, peptides were purchased from Synpeptide Co. and Genscript Inc. Peptides were first dissolved in DMSO to obtain a 10 mM stock solution and then diluted with distilled water to different concentrations before infiltration into *N. benthamiana* leaves.

### Western blot analysis

Protein extraction buffer (10 mM Tris/Cl [pH 7.5], 150 mM NaCl, 0.5 mM EDTA, 0.5% NP-40) supplemented with protease inhibitor cocktail (Roche) was used for protein extraction from mycelia and plant materials. Protein samples of *U. virens* mycelia from the WT strain and the overexpression transformants were collected 7 days after culturing in potato sucrose (PS) liquid medium. Western blot analysis was performed with the eBlot™ L1 Protein Transfer System (GenScript) and Odyssey (Li-COR) detection system with IRDye 800CW Goat anti-Mouse IgG(H + L) antibody (926-32210, LI-COR,1:10000). Chemiluminescence detection in Fig. 4b was carried out with Horseradish Peroxidase (HRP)-conjugated Anti- HA antibodies (26183-HRP, Thermo scientific, 1:2000). For detection using this system, the membrane was first incubated with developing reagents (SuperSignal West Pico & West Femto), and then imaged using ImageQuant LAS 4000 (Life Sciences). Anti-HA-tag primary monoclonal antibody (M20003L, Abmart, 1:3000) was used to detect the expression of proteins in *N. benthamiana* leaves. Anti-GFP-tag primary monoclonal antibody (M20004L, Abmart, 1:3000) was used to detect the expression of SGP1 and its mutants in *U. virens*.

### Evans blue staining

Evans Blue staining was performed to detect cell death in rice suspension cells^63^. This dye stains dead cells blue but does not stain living cells. The amount of Evans Blue released from rice suspension cells after staining was used as an indicator of cell death. Rice suspension cells were incubated with purified SGP1rec or MBP for 12 h followed by staining with 0.05% Evans Blue (Sigma) for 15 min. After extensive washing with water to remove excess dye, the blue dye taken up by dead cells was solubilized in 50% methanol with 1% SDS for 30 min at 50 °C and quantified by measuring light absorbance at 595 nm.

### VIGS assays in *N. benthamiana*

pTRV1 and pTRV2:BAK1 plasmid constructs were introduced into *Agrobacterium tumefaciens* GV3101 by electroporation. The cultured *Agrobacterium* cells were harvested and resuspended in infiltration buffer (10 mM MgCl_2_, 10 mM MES, pH 5.7, and 150 mM acetosyringone) to an OD of 0.5. An *Agrobacterium* strain harboring the pTRV2:BAK1 vector and one harboring the pTRV1 vector were mixed in a 1:1 ratio and left in the dark at 28 °C for 2 h before infiltration into the lower leaf of a four-leaf stage *N. benthamiana* plant using a 1 mL needleless syringe. pTRV2:EV was used as a negative control, and the phytoene desaturase gene (PDS) was used to evaluate the effectiveness of the VIGS assay. At about 3 weeks post inoculation of pTRV strains, the third new leaves were collected for RNA extraction and RT-PCR analysis to validate the silencing efficiency. These leaves were albino in the TRV:PDS lines, and thus should exhibit silencing of the BAK1 gene in the TRV:BAK1 lines. *Agrobacterium* strains harboring elicitors were then infiltrated into the third new leaves of VIGS-silenced plants.

### Phase partition assay and PI-PLC treatment

An HA tag was fused to the C terminus of the signal peptide and the N terminus of the mature SGP1 protein (SP-HA-mSGP1). The protein was expressed in *N. benthamiana* by *Agrobacterium*-mediated transient expression. Leaves were collected 2 days post *Agro*-infiltration. The protein was extracted with a homogenization buffer. After centrifugation for 10 min at 1200 × *g* and 4 °C, the post-nuclear supernatant was collected and phase partitioned with 2% (v/v) TX-114 according to a detailed protocol described previously^64^ with minor modifications. The detergent phase was washed with Tris-buffered saline (TBS) three times and then incubated in the absence or presence of 2 U/mL PI-PLC for 1 h at 37 °C. The final aqueous and detergent fractions were analyzed by western blot with anti-HA antibody (M20003L, Abmart, 1:3000). To further test whether GPI modification was involved in the cell death activity of SGP1, PI-PLC (2 U/mL) was infiltrated into *N. benthamiana* leaves 24 h after *Agro*-infiltration, or PI-PLC was infiltrated into *N. benthamiana* leaves together with SGP1rec.

### Measurement of reactive oxygen species

Reactive oxygen species (ROS) production was monitored with a luminol/peroxidase-based assay on leaf discs collected from 3- to 4-week-old *N. benthamiana* plants. The leaf discs were floated overnight in 200 μL of sterile H_2_O in a 96-well plate. H_2_O was replaced with working solution [luminol (35.4 μg/mL)/peroxidase (10 μg/mL) reaction solution supplied with sterile water and peptides at appropriate concentrations]. After the addition of the working solution, the plate was immediately moved to a GLOMAX96 microplate luminometer (Promega, Madison, WI, USA) for measurement of luminescence^24^.

### RNA extraction and RT-PCR analysis

Total RNA was extracted using a NucleoSpin RNA II kit (Invitrogen) following the manufacturer’s instructions and treated with DNase I (TaKaRa) to remove DNA contamination. Approximately 800 ng of RNA was used for reverse transcription with oligo(dT) primers. Real-time PCR was performed using an SYBR Premix Ex Taq Kit II (TaKaRa) according to the manufacturer’s protocol. The relative quantitative method (2^−ΔΔCt^) was used to evaluate the quantitative variation^65^. The α-*tubulin*, *NbEF1α*, and *Actin* genes were used as internal controls for *U. virens*, *N. benthamiana*, and rice samples, respectively.

### MAPK assays

Proteins were extracted from rice leaves and panicles after treatment with synthesized peptides using a plant phosphorylated protein extraction kit (HR0011, Baiaolaibo) following the manufacturer’s instructions. Phosphorylation of MAPK proteins was detected by immunoblotting with anti‐pMAPK antibody (#4370 s, Cell Signaling, 1:1000) and IRDye 800CW Goat anti-Rabbit IgG(H + L) antibody (926-32211, LI-COR, 1:10,000) according to the manufacturer’s protocol. Blots were stained with Ponceau S to verify equal loading.

### DAB staining

*N. benthamiana* leaves were infiltrated with different peptides at a concentration of 1 μM. At 6 and 12 hpi, the infiltrated leaves were stained with 3,3-Diaminobenzidine (DAB, D8001, Sigma) solution for 8 h in the dark and then destained with ethanol before observation. Similarly, rice leaves and panicles incubated with different peptides were also stained with DAB at 6 or 12 hpi.

### Culturing of microbial strains and preparation of pathogen inocula

Colonies of the *U. virens* WT strain and the transformants were grown on potato sucrose agar (PSA) medium plates. For conidia production, strains were cultured in PS liquid medium at 28 °C, with shaking at 150 rpm for 7 d. The cultures were then homogenized in a blender and adjusted to a concentration of about 10^6^ conidia/mL for use as inoculum on rice. To prepare zoospores of *P. parasitica*, mycelia were cultured in 2.5% liquid V8 juice medium for 3 days and repeatedly washed with sterilized water at RT and incubated at 25 °C until sporangia formed. To initiate zoospore release, fresh cold water (4 °C) was added into the plates, which were then incubated at 4 °C. *M. oryzae* strains were cultured on complete medium agar plates at 28 °C. For conidia production of *M. oryzae*, strains were maintained on straw decoction and corn agar media at 28 °C for 7 days in the dark followed by 3 days of continuous illumination under fluorescent light^66^. Then, about 20 mL of sterilized water was poured into the plate and the conidia were collected using a paint brush. The conidia suspension was filtered through three layers of lens paper, and resuspended to a concentration of 5 × 10^4^ spores/mL in sterile water. *Xoo* and *B. glumae* strains were grown on nutrient agar plates and King’s B medium plates at 28 °C for 2 days. Colonies were removed from the plate, washed twice, and resuspended to OD_600_ = 0.8 in sterile distilled water for inoculation on rice.

### Transformation of *U. virens*

The constructs for silencing the *SGP1* gene or overexpressing SGP1 and its mutants were transformed into *A. tumefaciens* strain AGL-1 and then transformed into conidia of the *U. virens* WT strain using the ATMT method^67–69^. In brief, 100 μL of *U. virens* conidia (10^6^ spores/mL) and 100 μL of *A. tumefaciens* strain AGL-1 containing the appropriate vector (OD600 was about 0.25) were mixed and spread on a nitrocellulose membrane. The nitrocellulose membrane was placed on a co-cultivation medium^69^ plate for 3 days and then transferred onto 2% TB3 [0.3% yeast extract, 0.3% casamino acid, 1% glucose, 2% sucrose (w/v)], amended with hygromycin B (100 μg/mL) as a selection agent for transformants and cefotaxime (800 μg/mL) to counter-select bacteria. After incubation at 28 °C for 5–6 days, individual transformant colonies appeared and were transferred to PSA plates containing 100 μg/mL hygromycin B for the second round of selection. The silencing transformants were screened by qRT-PCR with primers SGP1-RT-F/R and UvTubulin1-F/R. UvTubulin1 was used to normalize the expression of *SGP1*. The transformants overexpressing SGP1 were confirmed by western blot analysis with GFP antibody.

### Pathogenicity assays

For assaying the pathogenicity of *U. virens*, during the booting stage (5–7 days before heading), 2 mL of inoculum was injected into the upper parts (5–7 cm from the upper node) of the swollen sheaths, which enclosed the developing panicles, of flag leaves on the main stem or main tiller using a syringe. The inoculum was injected into the space between the sheath and panicle to allow the inoculum to surround the spikelets. Disease symptoms were observed 30 days after inoculation. For inducing PR gene expression in rice using *U. virens*, detached leaves and panicles were immersed in the *U. virens* conidia for 6 h and then kept wet in a petri dish containing wet filter paper and incubated at 28 °C for another 6 h. For assaying the disease resistance triggered by synthesized peptides, *N. benthamiana* leaves, rice panicles, and rice leaves were pretreated with 1 μM peptide for 24 h before pathogen inoculation. The peptide was infiltrated into *N. benthamiana* leaves with a needleless syringe and, in rice, was syringe injected into the swollen sheaths of flag leaves on the main stems or main tillers, or was sprayed onto leaves. For *P. parasitica* inoculation, the detached leaves of 4-week-old *N. benthamiana* plants were inoculated with 100 zoospores of *P. parasitica* and kept on wet paper in a petri dish for 2 days in the dark at 25 °C. For *M. oryzae* inoculation, two-week-old rice seedlings were sprayed with 5 ×10^4^/mL spores of *M. oryzae* strain Guy11 and kept wet at 28 °C. Disease symptoms were observed 7 days after inoculation. The number of each type of lesion (0, no lesion; Type 1, pinhead-sized brown specks; Type 2, 1.5-mm brown spots; Type 3, 2–3-mm gray spots with brown margins; Type 4, many elliptical gray spots longer than 3 mm; Type 5, coalesced lesions infecting 50% or more of the leaf area) per 5 cm of leaf strip was scored. To determine the relative pathogen biomass in *N. benthamiana*, rice spikelets, and rice leaves, samples were collected at 24 hpi, 5 dpi, and 3 dpi, respectively. For *Xoo* inoculation on rice, rice leaves were cut with sterile scissors that had been dipped in a bacterial suspension of *Xoo*. For *B. glumae* inoculation on rice panicles, 2 ml of *B. glumae* inoculum was inoculated onto rice panicles in the same way as described for *U. virens* inoculation. Disease symptoms were measured at 7 dpi. The disease severity in rice panicles infected by *B. glumae* was evaluated using the following scale: 0, healthy panicle; 1, 0–20% diseased panicle; 2, 21–40% diseased panicle; 3, 41–60% diseased panicle; 4, 61–80% diseased panicle; 5, 81–100% diseased panicle. Disease severity was calculated using the following formula: disease severity = Σ (number of samples per rating × rating value)/total number of panicles.

### RNA sequencing profiling

The mock- or SNP22-treated rice leaves and panicles were collected in three biological replicates. Samples from three biological replicates of the same treatment were pooled for RNA extraction and library preparation. After sequencing, clean reads were aligned to the *Oryza sativa Japonica* genome IRGSP-1.0 (Phytozome, http://www.phytozome.net) using TopHat version 2.1.1 with default parameters. Clean reads and total mapped reads for each sample were shown on Supplementary Data 10. Based on the length of each gene and the number of reads uniquely mapped to that gene, gene expression levels were normalized as fragments per kilobase of exon per million fragments mapped (FPKM) using Cufflinks version 2.2.1. To identify differentially expressed genes, a cutoff of |log2 fold change| (after adding “1” to each value in the matrix to avoid undefined values) > 2 was applied. The raw RNA-seq data have been deposited in the NCBI Sequence Read Archive data repository under accession number PRJNA690691.

### Confocal microscopy

Conidia and mycelia were visualized using a confocal microscope (LSM 710; Carl Zeiss).

### Statistics and reproducibility

SPSS statistics, GraphPad Prism, and Seaborn were used for data analysis. Similar results were obtained from three independent experiments in Figs. 1d, 7c, and 8c as well as in Supplementary Figs. 1, 3a, and 11c, d.

### Reporting summary

Further information on research design is available in the Nature Research Reporting Summary linked to this article.

## Supplementary information

Supplementary Information

Descriptions of Additional Supplementary Files

Supplementary Data 1

Supplementary Data 2

Supplementary Data 3

Supplementary Data 4

Supplementary Data 5

Supplementary Data 6

Supplementary Data 7

Supplementary Data 8

Supplementary Data 9

Supplementary Data 10

Reporting Summary

## Data Availability

The data that support the findings of this study are available in the manuscript and its supplementary files or are available from the corresponding authors upon reasonable request. RNA-seq data generated in this study are accessible in the NCBI Sequence Read Archive data repository under the accession number PRJNA690691. Proteomics data in this study are accessible in the Proteome Exchange partner repository under the accession number PXD02454. Source data are provided with this paper.

## Source data

Source Data

## References

[1] Nurnberger T, Brunner F (2002). Innate immunity in plants and animals: emerging parallels between the recognition of general elicitors and pathogen-associated molecular patterns. Curr. Opin. Plant Biol..

[2] Medzhitov R, Janeway CA (1997). Innate immunity: the virtues of a nonclonal system of recognition. Cell.

[3] Thomma BP, Nurnberger T, Joosten MH (2011). Of PAMPs and effectors: the blurred PTI-ETI dichotomy. Plant Cell.

[4] Boller T, Felix G (2009). A renaissance of elicitors: perception of microbe-associated molecular patterns and danger signals by pattern-recognition receptors. Annu. Rev. plant Biol..

[5] Zipfel C (2014). Plant pattern-recognition receptors. Trends Immunol..

[6] Gust AA (2007). Bacteria-derived peptidoglycans constitute pathogen-associated molecular patterns triggering innate immunity in *Arabidopsis*. J. Biol. Chem..

[7] Dow M, Newman MA, von Roepenack E (2000). The induction and modulation of plant defense responses by bacterial lipopolysaccharides. Annu. Rev. Phytopathol..

[8] Fliegmann J, Mithofer A, Wanner G, Ebel J (2004). An ancient enzyme domain hidden in the putative beta-glucan elicitor receptor of soybean may play an active part in the perception of pathogen-associated molecular patterns during broad host resistance. J. Biol. Chem..

[9] Shinya T, Nakagawa T, Kaku H, Shibuya N (2015). Chitin-mediated plant-fungal interactions: catching, hiding and handshaking. Curr. Opin. Plant Biol..

[10] Felix G, Duran JD, Volko S, Boller T (1999). Plants have a sensitive perception system for the most conserved domain of bacterial flagellin. Plant J..

[11] Kunze G (2004). The N terminus of bacterial elongation factor Tu elicits innate immunity in *Arabidopsis* plants. Plant Cell.

[12] Zipfel C (2006). Perception of the bacterial PAMP EF-Tu by the receptor EFR restricts *Agrobacterium*-mediated transformation. Cell.

[13] Felix G, Boller T (2003). Molecular sensing of bacteria in plants. The highly conserved RNA-binding motif RNP-1 of bacterial cold shock proteins is recognized as an elicitor signal in tobacco. J. Biol. Chem..

[14] Brunner F (2002). Pep-13, a plant defense-inducing pathogen-associated pattern from *Phytophthora* transglutaminases. EMBO J..

[15] Gaulin E (2006). Cellulose binding domains of a *Phytophthora* cell wall protein are novel pathogen-associated molecular patterns. Plant Cell.

[16] Yu LM (1995). Elicitins from *Phytophthora* and basic resistance in tobacco. Proc. Natl Acad. Sci. USA.

[17] Rotblat B, Enshell-Seijffers D, Gershoni JM, Schuster S, Avni A (2002). Identification of an essential component of the elicitation active site of the EIX protein elicitor. Plant J..

[18] Qutob D (2006). Phytotoxicity and innate immune responses induced by Nep1-like proteins. Plant Cell.

[19] Oome S (2014). Nep1-like proteins from three kingdoms of life act as a microbe-associated molecular pattern in *Arabidopsis*. Proc. Natl Acad. Sci. USA.

[20] Bohm H (2014). A conserved peptide pattern from a widespread microbial virulence factor triggers pattern-induced immunity in *Arabidopsis*. PLoS Pathog..

[21] Albert I (2015). An RLP23-SOBIR1-BAK1 complex mediates NLP-triggered immunity. Nat. Plants.

[22] Gijzen M, Nurnberger T (2006). Nep1-like proteins from plant pathogens: recruitment and diversification of the NPP1 domain across taxa. Phytochemistry.

[23] Ma Z (2015). A *Phytophthora sojae* glycoside hydrolase 12 protein is a major virulence factor during soybean infection and is recognized as a PAMP. Plant Cell.

[24] Wang Y (2018). Leucine-rich repeat receptor-like gene screen reveals that *Nicotiana* RXEG1 regulates glycoside hydrolase 12 MAMP detection. Nat. Commun..

[25] Monaghan J, Zipfel C (2012). Plant pattern recognition receptor complexes at the plasma membrane. Curr. Opin. Plant Biol..

[26] Bohm H, Albert I, Fan L, Reinhard A, Nurnberger T (2014). Immune receptor complexes at the plant cell surface. Curr. Opin. Plant Biol..

[27] Zurzolo C, Simons K (2016). Glycosylphosphatidylinositol-anchored proteins: membrane organization and transport. Biochim. Biophys. Acta.

[28] Chatterjee S, Mayor S (2001). The GPI-anchor and protein sorting. Cell Mol. Life Sci..

[29] Kinoshita T, Fujita M, Maeda Y (2008). Biosynthesis, remodelling and functions of mammalian GPI-anchored proteins: recent progress. J. Biochem..

[30] Paulick MG, Bertozzi CR (2008). The glycosylphosphatidylinositol anchor: a complex membrane-anchoring structure for proteins. Biochemistry.

[31] Griffith OH, Ryan M (1999). Bacterial phosphatidylinositol-specific phospholipase C: structure, function, and interaction with lipids. Biochim. Biophys. Acta.

[32] Yeats TH, Bacic A, Johnson KL (2018). Plant glycosylphosphatidylinositol anchored proteins at the plasma membrane-cell wall nexus. J. Integr. Plant Biol..

[33] Tang Y-X (2012). Elucidation of the infection process of *Ustilaginoidea virens* (teleomorph: *Villosiclava virens*) in rice spikelets. Plant Pathol..

[34] Rush MC, Shahjahan AKM, Jones JP, Groth DE (2000). Outbreak of false smut of rice in Louisiana. Plant Dis..

[35] Jiehua Q, Shuai M, Yizhen D, Shiwen H, Yanjun K (2019). *Ustilaginoidea virens*: a fungus infects rice flower and threats world rice production. Rice Sci..

[36] Song JH (2016). Rice false smut fungus hijacks the rice nutrients supply by blocking and mimicking the fertilization of rice ovary. Environ. Microbiol.

[37] Hu M, Luo L, Wang S, Liu Y, Li J (2013). Infection processes of *Ustilaginoidea virens* during artificial inoculation of rice panicles. Eur. J. Plant Pathol..

[38] Ngugi HK, Scherm H (2006). Biology of flower-infecting fungi. Annu. Rev. Phytopathol..

[39] Ashizawa T, Kataoka Y (2005). Detection of *Ustilaginoidea virens* in rice panicles before and after heading in the field using nested-PCR technique with species-specific primers. Jpn. J. Phytopathol..

[40] Ashizawa T, Takahashi M, Arai M, Arie T (2012). Rice false smut pathogen, *Ustilaginoidea virens*, invades through small gap at the apex of a rice spikelet before heading. J. Gen. Plant Pathol..

[41] Meindl T, Boller T, Felix G (2000). The bacterial elicitor flagellin activates its receptor in tomato cells according to the address-message concept. Plant Cell.

[42] Hu H (2015). A receptor like kinase gene with expressional responsiveness on *Xanthomonas oryzae* pv. oryzae is essential for Xa21-mediated disease resistance. Rice.

[43] Westergard L, Christensen HM, Harris DA (2007). The cellular prion protein (PrP(C)): its physiological function and role in disease. Biochim. Biophys. Acta.

[44] Moore RA, Taubner LM, Priola SA (2009). Prion protein misfolding and disease. Curr. Opin. Struct. Biol..

[45] Scialo, C., De Cecco, E., Manganotti, P. & Legname, G. Prion and prion-like protein strains: deciphering the molecular basis of heterogeneity in neurodegeneration. *Viruses* **11**, 261 (2019). 10.3390/v11030261 PMC6466326 30875755

[46] Liu X (2016). The role of LORELEI in pollen tube reception at the interface of the synergid cell and pollen tube requires the modified eight-cysteine motif and the receptor-like kinase FERONIA. Plant Cell.

[47] Martinez-Lopez R, Monteoliva L, Diez-Orejas R, Nombela C, Gil C (2004). The GPI-anchored protein CaEcm33p is required for cell wall integrity, morphogenesis and virulence in *Candida albicans*. Microbiology.

[48] Chang PK (2018). *Aspergillus flavus* GPI-anchored protein-encoding ecm33 has a role in growth, development, aflatoxin biosynthesis, and maize infection. Appl. Microbiol. Biotechnol..

[49] Bailey BA, Dean JF, Anderson JD (1990). An ethylene biosynthesis-inducing endoxylanase elicits electrolyte leakage and necrosis in *Nicotiana tabacum* cv Xanthi leaves. Plant Physiol..

[50] Wei ZM (1992). Harpin, elicitor of the hypersensitive response produced by the plant pathogen *Erwinia amylovora*. Science.

[51] Khatib M, Lafitte C, Esquerre-Tugaye MT, Bottin A, Rickauer M (2004). The CBEL elicitor of *Phytophthora parasitica* var. nicotianae activates defence in *Arabidopsis thaliana* via three different signalling pathways. N. Phytol..

[52] Ron M, Avni A (2004). The receptor for the fungal elicitor ethylene-inducing xylanase is a member of a resistance-like gene family in tomato. Plant Cell.

[53] Raz E (2007). Organ-specific regulation of innate immunity. Nat. Immunol..

[54] Hu W, Pasare C (2013). Location, location, location: tissue-specific regulation of immune responses. J. Leukoc. Biol..

[55] Motoyama T (2002). Isolation and analysis of genes from phytopathogenic fungi. Prog. Biotechnol..

[56] Balmer D, de Papajewski DV, Planchamp C, Glauser G, Mauch-Mani B (2013). Induced resistance in maize is based on organ-specific defence responses. Plant J..

[57] Plancot B (2013). Deciphering the responses of root border-like cells of *Arabidopsis* and flax to pathogen-derived elicitors. Plant Physiol..

[58] Wyrsch I, Dominguez-Ferreras A, Geldner N, Boller T (2015). Tissue-specific FLAGELLIN-SENSING 2 (FLS2) expression in roots restores immune responses in *Arabidopsis* fls2 mutants. N. Phytol..

[59] Beck M (2014). Expression patterns of flagellin sensing 2 map to bacterial entry sites in plant shoots and roots. J. Exp. Bot..

[60] Emms DM, Kelly S (2015). OrthoFinder: solving fundamental biases in whole genome comparisons dramatically improves orthogroup inference accuracy. Genome Biol..

[61] Gouy M, Guindon S, Gascuel O (2010). SeaView version 4: a multiplatform graphical user interface for sequence alignment and phylogenetic tree building. Mol. Biol. Evol..

[62] Tamura K, Stecher G, Peterson D, Filipski A, Kumar S (2013). MEGA6: molecular evolutionary genetics analysis version 6.0. Mol. Biol. Evol..

[63] Fang-Sik Che MI (1999). Biochemical and morphological features of rice cell death induced by *Pseudomonas avenae*. Plant Cell Physiol..

[64] Bernat-Silvestre C (2020). p24 Family proteins are involved in transport to the plasma membrane of GPI-anchored proteins in plants. Plant Physiol..

[65] Kenneth J, Livaka TDS (2001). Analysis of relative gene expression data using real-time quantitative PCR and the 2−ΔΔCT method. Methods Mol. Biol..

[66] Guo M (2010). The basic leucine zipper transcription factor Moatf1 mediates oxidative stress responses and is necessary for full virulence of the rice blast fungus *Magnaporthe oryzae*. Mol. Plant Microbe Interact..

[67] Yu M (2015). Identification of pathogenicity-related genes in the rice pathogen *Ustilaginoidea virens* through random insertional mutagenesis. Fungal Genet. Biol..

[68] Yu J (2019). A homeobox transcription factor UvHOX2 regulates chlamydospore formation, conidiogenesis, and pathogenicity in *Ustilaginoidea virens*. Front. Microbiol..

[69] Mullins ED (2001). *Agrobacterium*-mediated transformation of *Fusarium oxysporum*: an efficient tool for insertional mutagenesis and gene transfer. Phytopathology.

