# Supplementary material for: The N-terminus of an Ustilaginoidea virens Ser-Thr-rich glycosylphosphatidylinositol-anchored protein elicits plant immunity as a MAMP
Source: Nat Commun. 2021 Apr 27;12:2451. doi: 10.1038/s41467-021-22660-9 (PMC8079714; doi:10.1038/s41467-021-22660-9)
Supplement: Supplementary file 1 — Supplementary Information [file 41467_2021_22660_MOESM1_ESM.pdf]

Supplementary Information:

The N-terminus of an *Ustilaginoidea virens* Ser-Thr-rich glycosylphosphatidylinositol-anchored protein elicits plant immunity as a MAMP

Song et al.

Contents:

Supplementary Figures

Supplementary Fig. 1 SDS-PAGE of recombinant fusion proteins purified from *E. coli*.
Supplementary Fig. 2 Illustration of the cell death index.
Supplementary Fig. 3 Biochemical characterization of SGP1.
Supplementary Fig. 4 SNPrec-triggered cell death is not affected by heat treatment.
Supplementary Fig. 5 ClustalW alignment of 124 protein sequences that contain the Ser-Thr-rich Glycosyl-phosphatidyl-inositol-anchored domain.
Supplementary Fig. 6 Cell death responses induced by synthesized peptides.
Supplementary Fig. 7 The I25A and W44A mutations attenuate the ability of SGP1 to induce cell death and ROS production.
Supplementary Fig. 8 The synthesized peptides do not affect filamentous growth of pathogens on plates.
Supplementary Fig. 9 Activation of immunity in *N. benthamiana* triggered by peptides.
Supplementary Fig. 10 Activation of immunity in rice triggered by elicitors.
Supplementary Fig. 11 Silencing of SGP1 or overexpression of SGP1 or SGP1^{W44A} does not affect *U. virens* filamentous growth, conidia morphology, or hyphal morphology.

Supplementary figures

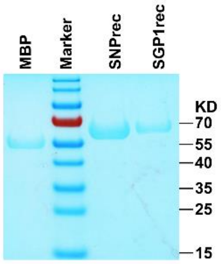

Supplementary Fig. 1 SDS-PAGE of recombinant fusion proteins purified from *E. coli.* Mature sequences of SGP1 and the N-terminus of SGP1 (SNP, residues 18–120), where the Ser-Thr-rich GPI-anchored domain is located, were fused in frame with MBP at the C-terminus.

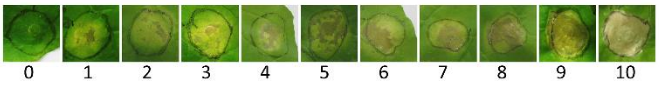

Supplementary Fig. 2 Illustration of the cell death index. Images showing cell death phenotypes at scales ranging from 0 (no cell death phenotype) to 10 (fully confluent necrosis) are shown.

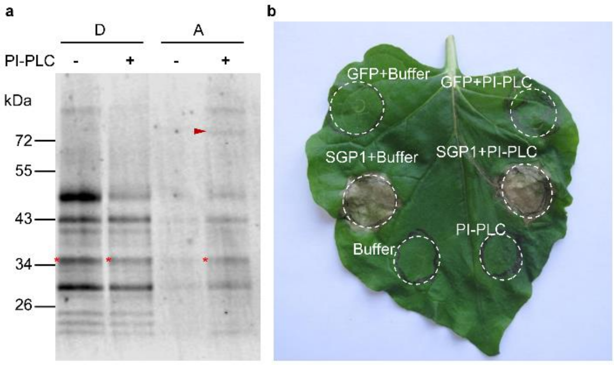

Supplementary Fig. 3 Biochemical characterization of SGP1. a, SGP1 can be released from the detergent phase to aqueous phase by treatment with Phosphatidyl-Inositol-Phospholipase C (PI-PLC). Post-nuclear supernatants from *N. benthamiana* leaves expressing SP-HA-SGP1 were extracted with Triton X-114, and the detergent phase was subjected to a mock treatment (-) or treatment with of PI-PLC (+). After the treatment, the detergent (D) and aqueous (A) phases were separated and analyzed by western blot with HA antibodies. **b,** Cell death activity of SGP1 in the presence or absence of PI-PLC. PI-PLC was infiltrated 24 h after *Agrobacterium* infiltration. Three independent experiments were performed; in each experiment there were three leaves per treatment.

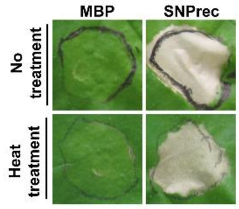

Supplementary Fig. 4 SNPrec-triggered cell death is not affected by heat treatment. Cell death triggered by heat-treated SNP recombinant protein (SNPrec). SNPrec was heat-treated by boiling for 10 min at 100°C. Representative images of cell death response in *N. benthamiana* leaves taken at 5 dpi. Three independent experiments were performed as shown in Supplementary Data 3. For each experiment, there were ten leaves per treatment.

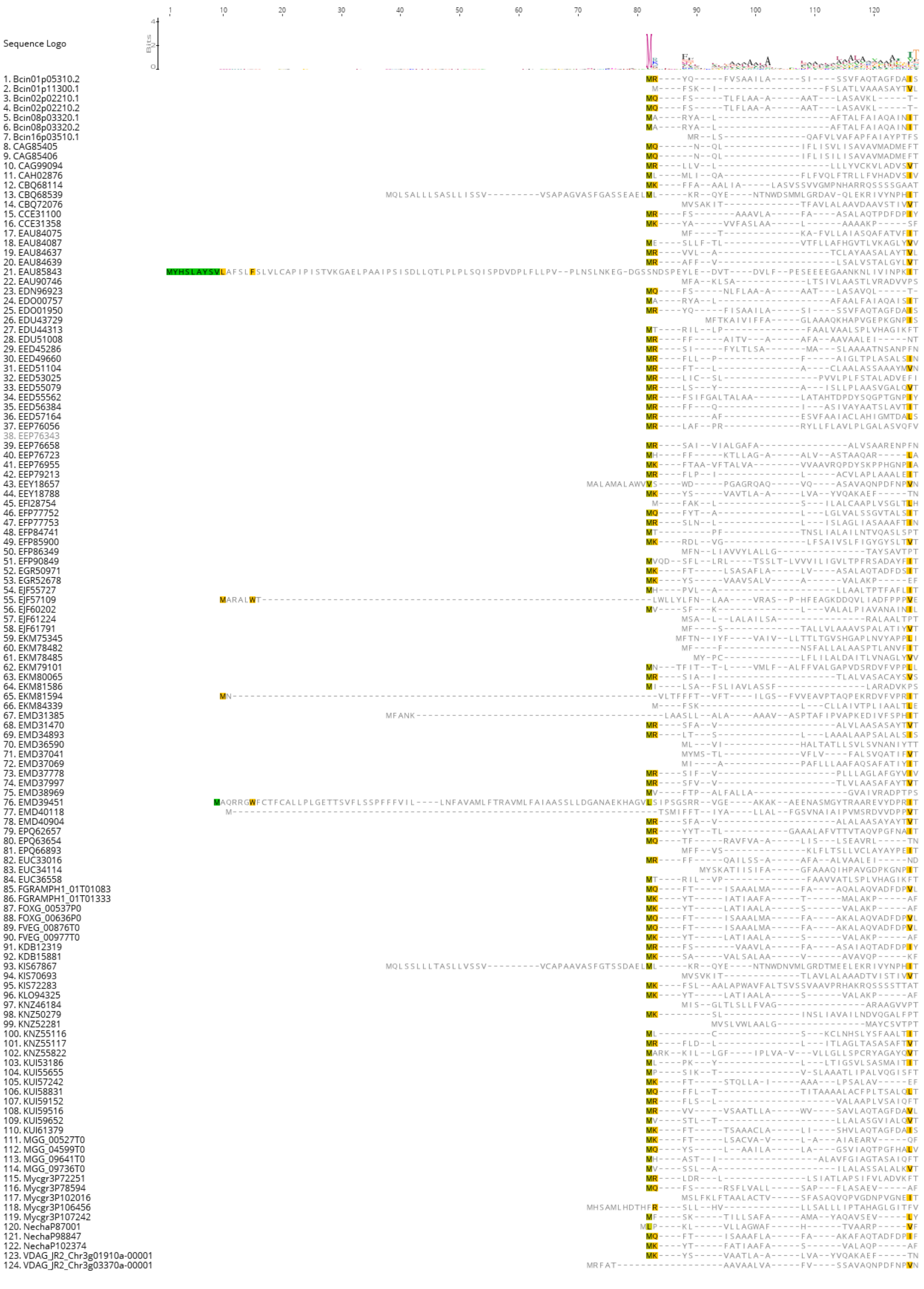
Sequence Logo
Bits
1. Bcin01p05310.2
2. Bcin01p11300.1
3. Bcin02p02210.1
4. Bcin02p02210.2
5. Bcin08p03320.1
6. Bcin08p03320.2
7. Bcin16p03510.1
8. CAG85405
9. CAG85406
10. CAG99094
11. CAH02876
12. CBQ68114
13. CBQ68539
14. CBQ72076
15. CCE31100
16. CCE31358
17. EAU84075
18. EAU84087
19. EAU84637
20. EAU84639
21. EAU85843
22. EAU90746
23. EDN96923
24. EDO00757
25. EDO01950
26. EDU43729
27. EDU44313
28. EDU51008
29. EED45286
30. EED49660
31. EED51104
32. EED53025
33. EED55079
34. EED55562
35. EED56384
36. EED57164
37. EEP76056
38. EEP76343
39. EEP76658
40. EEP76723
41. EEP76955
42. EEP79213
43. EEY18657
44. EEY18788
45. EFI28754
46. EFP77752
47. EFP77753
48. EFP84741
49. EFP85900
50. EFP86349
51. EFP90849
52. EGR50971
53. EGR52678
54. EJF55727
55. EJF57109
56. EJF60202
57. EJF61224
58. EJF61791
59. EKM75345
60. EKM78482
61. EKM78485
62. EKM79101
63. EKM80065
64. EKM81586
65. EKM81594
66. EKM84339
67. EMD31385
68. EMD31470
69. EMD34893
70. EMD36590
71. EMD37041
72. EMD37069
73. EMD37778
74. EMD37997
75. EMD38969
76. EMD39451
77. EMD40118
78. EMD40904
79. EPQ62657
80. EPQ63654
81. EPQ66893
82. EUC33016
83. EUC34114
84. EUC36558
85. FGRAMPH1_01T01083
86. FGRAMPH1_01T01333
87. FOXG_00537P0
88. FOXG_00636P0
89. FVEG_00876T0
90. FVEG_00977T0
91. KDB12319
92. KDB15881
93. KIS67867
94. KIS70693
95. KIS72283
96. KLO94325
97. KNZ46184
98. KNZ50279
99. KNZ52281
100. KNZ55116
101. KNZ55117
102. KNZ55822
103. KUI53186
104. KUI55655
105. KUI57242
106. KUI58831
107. KUI59152
108. KUI59516
109. KUI59652
110. KUI61379
111. MGG_00527T0
112. MGG_04599T0
113. MGG_09641T0
114. MGG_09736T0
115. Mycgr3P72251
116. Mycgr3P78594
117. Mycgr3P102016
118. Mycgr3P106456
119. Mycgr3P107242
120. NechaP87001
121. NechaP98847
122. NechaP102374
123. VDAG_JR2_Chr3g01910a-00001
124. VDAG_JR2_Chr3g03370a-00001

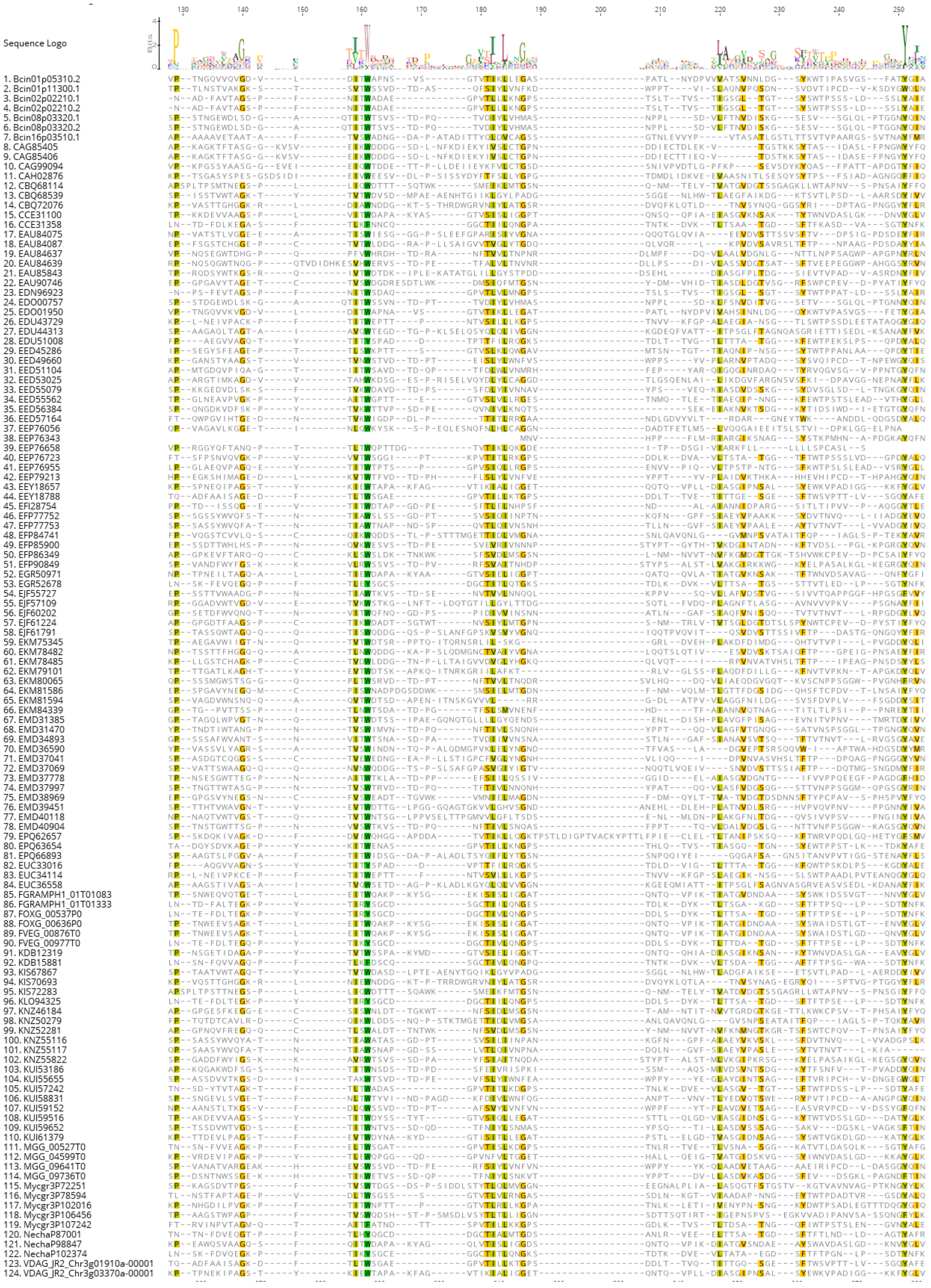

130
140
150
160
170
180
190
200
210
220
230
240
250
Sequence Logo
Bits
1. Bcin01p05310.2
2. Bcin01p11300.1
3. Bcin02p02210.1
4. Bcin02p02210.2
5. Bcin08p03320.1
6. Bcin08p03320.2
7. Bcin16p03510.1
8. CAG85405
9. CAG85406
10. CAG99094
11. CAH02876
12. CBQ68114
13. CBQ68539
14. CBQ72076
15. CCE31100
16. CCE31358
17. EAU84075
18. EAU84087
19. EAU84637
20. EAU84639
21. EAU85843
22. EAU90746
23. EDN96923
24. EDO00757
25. EDO01950
26. EDU43729
27. EDU44313
28. EDU51008
29. EED45286
30. EED49660
31. EED51104
32. EED53025
33. EED55079
34. EED55562
35. EED56384
36. EED57164
37. EEP76056
38. EEP76343
39. EEP76658
40. EEP76723
41. EEP76955
42. EEP79213
43. EEY18657
44. EEY18788
45. EFI28754
46. EFP77752
47. EFP77753
48. EFP84741
49. EFP85900
50. EFP86349
51. EFP90849
52. EGR50971
53. EGR52678
54. EJF55727
55. EJF57109
56. EJF60202
57. EJF61224
58. EJF61791
59. EKM75345
60. EKM78482
61. EKM78485
62. EKM79101
63. EKM80065
64. EKM81586
65. EKM81594
66. EKM84339
67. EMD31385
68. EMD31470
69. EMD34893
70. EMD36590
71. EMD37041
72. EMD37069
73. EMD37778
74. EMD37997
75. EMD38969
76. EMD39451
77. EMD40118
78. EMD40904
79. EPQ62657
80. EPQ63654
81. EPQ66893
82. EUC33016
83. EUC34114
84. EUC36558
85. FGRAMPH1_01T01083
86. FGRAMPH1_01T01333
87. FOXG_00537P0
88. FOXG_00636P0
89. FVEG_00876T0
90. FVEG_00977T0
91. KDB12319
92. KDB15881
93. KIS67867
94. KIS70693
95. KIS72283
96. KLO94325
97. KNZ46184
98. KNZ50279
99. KNZ52281
100. KNZ55116
101. KNZ55117
102. KNZ55822
103. KUI53186
104. KUI55655
105. KUI57242
106. KUI58831
107. KUI59152
108. KUI59516
109. KUI59652
110. KUI61379
111. MGG_00527T0
112. MGG_04599T0
113. MGG_09641T0
114. MGG_09736T0
115. Mycgr3P72251
116. Mycgr3P78594
117. Mycgr3P102016
118. Mycgr3P106456
119. Mycgr3P107242
120. NechaP87001
121. NechaP98847
122. NechaP102374
123. VDAG_JR2_Chr3g01910a-00001
124. VDAG_JR2_Chr3g03370a-00001

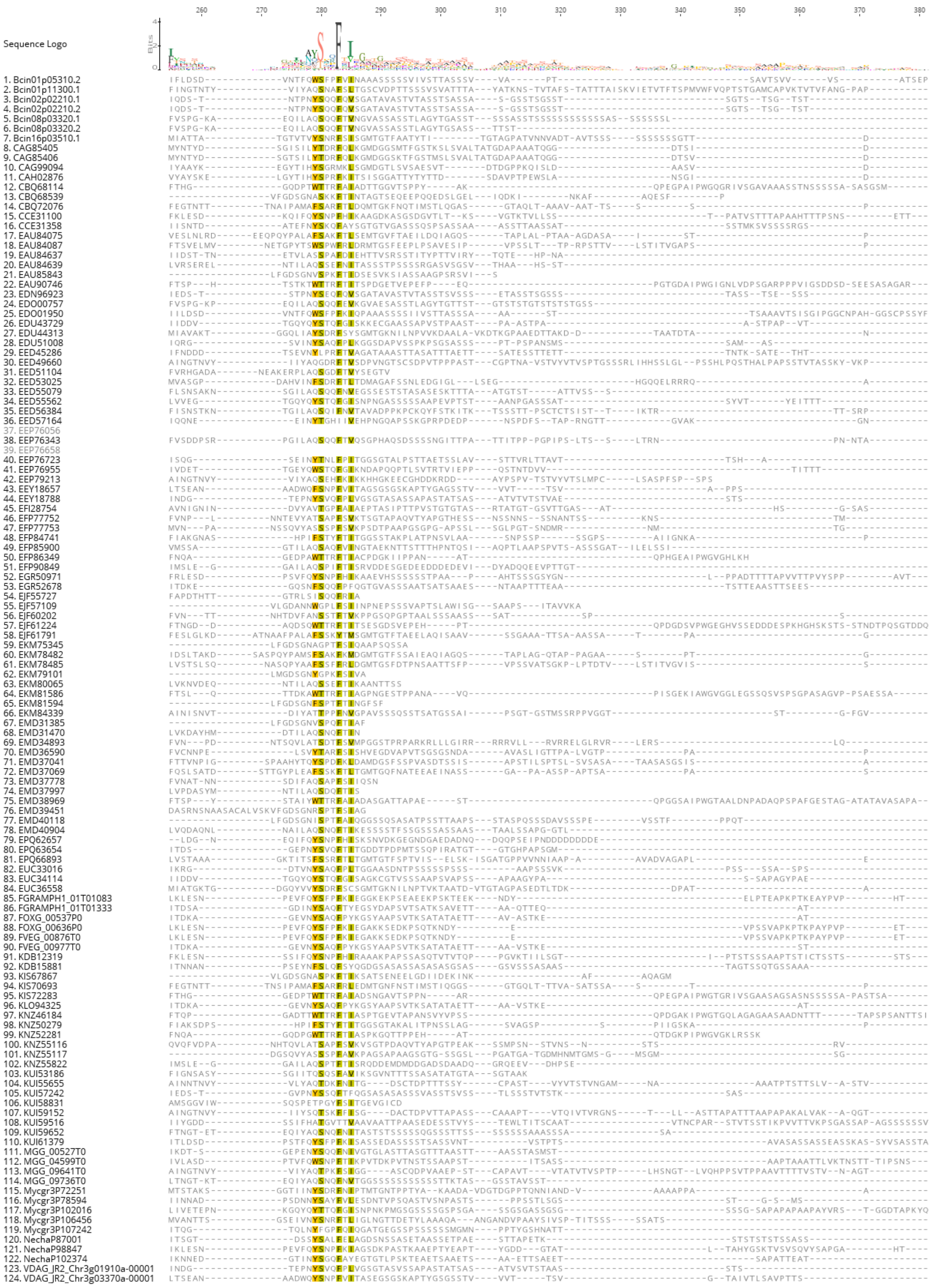

260
270
280
290
300
310
320
330
340
350
360
370
380
Sequence Logo
Bits
1. Bcin01p05310.2
2. Bcin01p11300.1
3. Bcin02p02210.1
4. Bcin02p02210.2
5. Bcin08p03320.1
6. Bcin08p03320.2
7. Bcin16p03510.1
8. CAG85405
9. CAG85406
10. CAG99094
11. CAH02876
12. CBQ68114
13. CBQ68539
14. CBQ72076
15. CCE31100
16. CCE31358
17. EAU84075
18. EAU84087
19. EAU84637
20. EAU84639
21. EAU85843
22. EAU90746
23. EDN96923
24. EDO00757
25. EDO01950
26. EDU43729
27. EDU44313
28. EDU51008
29. EED45286
30. EED49660
31. EED51104
32. EED53025
33. EED55079
34. EED55562
35. EED56384
36. EED57164
37. EEP76056
38. EEP76343
39. EEP76658
40. EEP76723
41. EEP76955
42. EEP79213
43. EEY18657
44. EEY18788
45. EFI28754
46. EFP77752
47. EFP77753
48. EFP84741
49. EFP85900
50. EFP86349
51. EFP90849
52. EGR50971
53. EGR52678
54. EJF55727
55. EJF57109
56. EJF60202
57. EJF61224
58. EJF61791
59. EKM75345
60. EKM78482
61. EKM78485
62. EKM79101
63. EKM80065
64. EKM81586
65. EKM81594
66. EKM84339
67. EMD31385
68. EMD31470
69. EMD34893
70. EMD36590
71. EMD37041
72. EMD37069
73. EMD37778
74. EMD37997
75. EMD38969
76. EMD39451
77. EMD40118
78. EMD40904
79. EPQ62657
80. EPQ63654
81. EPQ66893
82. EUC33016
83. EUC34114
84. EUC36558
85. FGRAMPH1_01T01083
86. FGRAMPH1_01T01333
87. FOXG_00537P0
88. FOXG_00636P0
89. FVEG_00876T0
90. FVEG_00977T0
91. KDB12319
92. KDB15881
93. KIS67867
94. KIS70693
95. KIS72283
96. KLO94325
97. KNZ46184
98. KNZ50279
99. KNZ52281
100. KNZ55116
101. KNZ55117
102. KNZ55822
103. KUI53186
104. KUI55655
105. KUI57242
106. KUI58831
107. KUI59152
108. KUI59516
109. KUI59652
110. KUI61379
111. MGG_00527T0
112. MGG_04599T0
113. MGG_09641T0
114. MGG_09736T0
115. Mycgr3P72251
116. Mycgr3P78594
117. Mycgr3P102016
118. Mycgr3P106456
119. Mycgr3P107242
120. NechaP87001
121. NechaP98847
122. NechaP102374
123. VDAG_JR2_Chr3g01910a-00001
124. VDAG_JR2_Chr3g03370a-00001

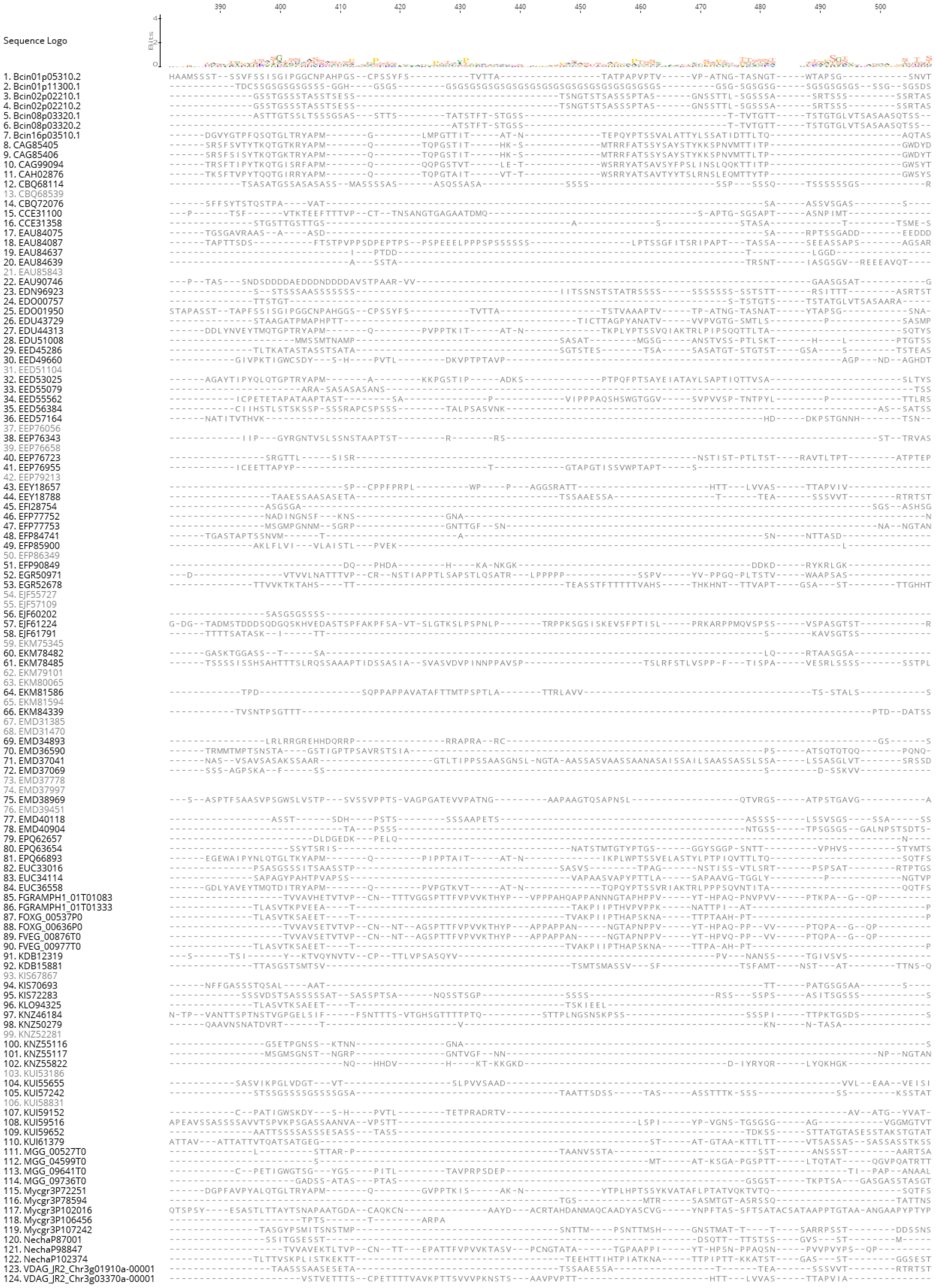
Sequence Logo
Bits
390
400
410
420
430
440
450
460
470
480
490
500
1. Bcin01p05310.2
2. Bcin01p11300.1
3. Bcin02p02210.1
4. Bcin02p02210.2
5. Bcin08p03320.1
6. Bcin08p03320.2
7. Bcin16p03510.1
8. CAG85405
9. CAG85406
10. CAG99094
11. CAH02876
12. CBQ68114
13. CBQ68539
14. CBQ72076
15. CCE31100
16. CCE31358
17. EAU84075
18. EAU84087
19. EAU84637
20. EAU84639
21. EAU85843
22. EAU90746
23. EDN96923
24. EDO00757
25. EDO01950
26. EDU43729
27. EDU44313
28. EDU51008
29. EED45286
30. EED49660
31. EED51104
32. EED53025
33. EED55079
34. EED55562
35. EED56384
36. EED57164
37. EEP76056
38. EEP76343
39. EEP76658
40. EEP76723
41. EEP76955
42. EEP79213
43. EEY18657
44. EEY18788
45. EFI28754
46. EFP77752
47. EFP77753
48. EFP84741
49. EFP85900
50. EFP86349
51. EFP90849
52. EGR50971
53. EGR52678
54. EJF55727
55. EJF57109
56. EJF60202
57. EJF61224
58. EJF61791
59. EKM75345
60. EKM78482
61. EKM78485
62. EKM79101
63. EKM80065
64. EKM81586
65. EKM81594
66. EKM84339
67. EMD31385
68. EMD31470
69. EMD34893
70. EMD36590
71. EMD37041
72. EMD37069
73. EMD37778
74. EMD37997
75. EMD38969
76. EMD39451
77. EMD40118
78. EMD40904
79. EPQ62657
80. EPQ63654
81. EPQ66893
82. EUC33016
83. EUC34114
84. EUC36558
85. FGRAMPH1_01T01083
86. FGRAMPH1_01T01333
87. FOXG_00537P0
88. FOXG_00636P0
89. FVEG_00876T0
90. FVEG_00977T0
91. KDB12319
92. KDB15881
93. KIS67867
94. KIS70693
95. KIS72283
96. KLO94325
97. KNZ46184
98. KNZ50279
99. KNZ52281
100. KNZ55116
101. KNZ55117
102. KNZ55822
103. KUI53186
104. KUI55655
105. KUI57242
106. KUI58831
107. KUI59152
108. KUI59516
109. KUI59652
110. KUI61379
111. MGG_00527T0
112. MGG_04599T0
113. MGG_09641T0
114. MGG_09736T0
115. Mycgr3P72251
116. Mycgr3P78594
117. Mycgr3P102016
118. Mycgr3P106456
119. Mycgr3P107242
120. NechaP87001
121. NechaP98847
122. NechaP102374
123. VDAG_JR2_Chr3g01910a-00001
124. VDAG_JR2_Chr3g03370a-00001

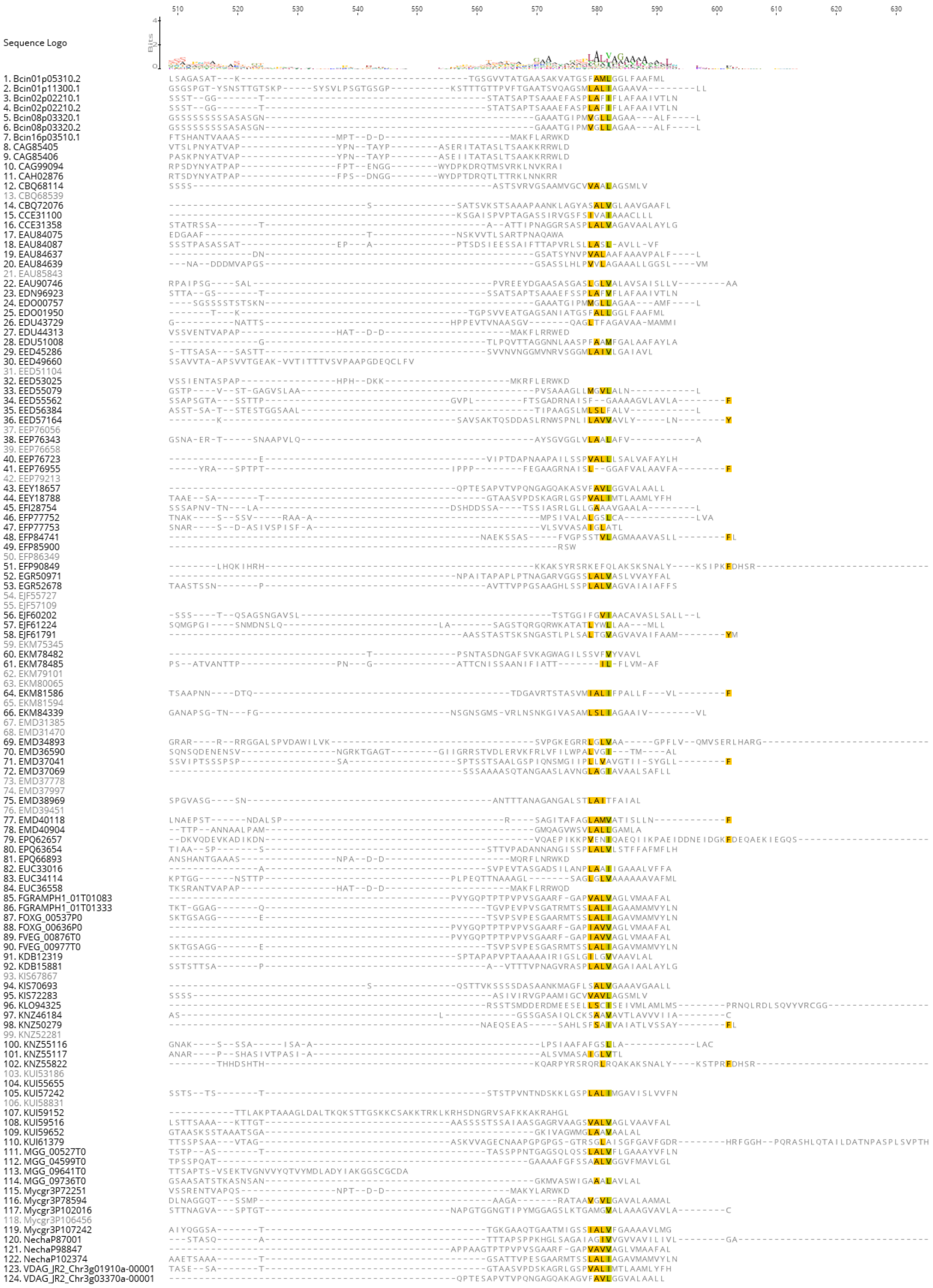

Sequence Logo
Bits
510
520
530
540
550
560
570
580
590
600
610
620
630
1. Bcin01p05310.2
2. Bcin01p11300.1
3. Bcin02p02210.1
4. Bcin02p02210.2
5. Bcin08p03320.1
6. Bcin08p03320.2
7. Bcin16p03510.1
8. CAG85405
9. CAG85406
10. CAG99094
11. CAH02876
12. CBQ68114
13. CBQ68539
14. CBQ72076
15. CCE31100
16. CCE31358
17. EAU84075
18. EAU84087
19. EAU84637
20. EAU84639
21. EAU85843
22. EAU90746
23. EDN96923
24. EDO00757
25. EDO01950
26. EDU43729
27. EDU44313
28. EDU51008
29. EED45286
30. EED49660
31. EED51104
32. EED53025
33. EED55079
34. EED55562
35. EED56384
36. EED57164
37. EEP76056
38. EEP76343
39. EEP76658
40. EEP76723
41. EEP76955
42. EEP79213
43. EEY18657
44. EEY18788
45. EFI28754
46. EFP77752
47. EFP77753
48. EFP84741
49. EFP85900
50. EFP86349
51. EFP90849
52. EGR50971
53. EGR52678
54. EJF55727
55. EJF57109
56. EJF60202
57. EJF61224
58. EJF61791
59. EKM75345
60. EKM78482
61. EKM78485
62. EKM79101
63. EKM80065
64. EKM81586
65. EKM81594
66. EKM84339
67. EMD31385
68. EMD31470
69. EMD34893
70. EMD36590
71. EMD37041
72. EMD37069
73. EMD37778
74. EMD37997
75. EMD38969
76. EMD39451
77. EMD40118
78. EMD40904
79. EPQ62657
80. EPQ63654
81. EPQ66893
82. EUC33016
83. EUC34114
84. EUC36558
85. FGRAMPH1_01T01083
86. FGRAMPH1_01T01333
87. FOXG_00537P0
88. FOXG_00636P0
89. FVEG_00876T0
90. FVEG_00977T0
91. KDB12319
92. KDB15881
93. KIS67867
94. KIS70693
95. KIS72283
96. KLO94325
97. KNZ46184
98. KNZ50279
99. KNZ52281
100. KNZ55116
101. KNZ55117
102. KNZ55822
103. KUI53186
104. KUI55655
105. KUI57242
106. KUI58831
107. KUI59152
108. KUI59516
109. KUI59652
110. KUI61379
111. MGG_00527T0
112. MGG_04599T0
113. MGG_09641T0
114. MGG_09736T0
115. Mycgr3P72251
116. Mycgr3P78594
117. Mycgr3P102016
118. Mycgr3P106456
119. Mycgr3P107242
120. NechaP87001
121. NechaP98847
122. NechaP102374
123. VDAG_JR2_Chr3g01910a-00001
124. VDAG_JR2_Chr3g03370a-00001

Sequence Logo

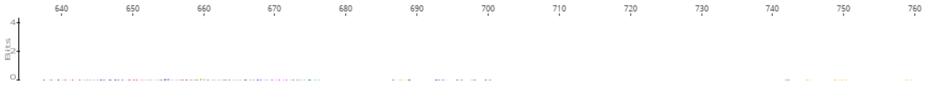

1. Bcin01p05310.2
2. Bcin01p11300.1
3. Bcin02p02210.1
4. Bcin02p02210.2
5. Bcin08p03320.1
6. Bcin08p03320.2
7. Bcin16p03510.1
8. CAG85405
9. CAG85406
10. CAG99094
11. CAH02876
12. CBQ68114
13. CBQ68539
14. CBQ72076
15. CCE31100
16. CCE31358
17. EAU84075
18. EAU84087
19. EAU84637
20. EAU84639
21. EAU85843
22. EAU90746
23. EDN96923
24. EDO00757
25. EDO01950
26. EDU43729
27. EDU44313
28. EDU51008
29. EED45286
30. EED49660
31. EED51104
32. EED53025
33. EED55079
34. EED55562
35. EED56384
36. EED57164
37. EEP76056
38. EEP76343
39. EEP76658
40. EEP76723
41. EEP76955
42. EEP79213
43. EEY18657
44. EEY18788
45. EFI28754
46. EFP77752
47. EFP77753
48. EFP84741
49. EFP85900
50. EFP86349
51. EFP90849 ---------------SACSRSPRNPKIAKIV
52. EGR50971
53. EGR52678
54. EJF55727
55. EJF57109
56. EJF60202
57. EJF61224
58. EJF61791
59. EKM75345
60. EKM78482
61. EKM78485
62. EKM79101
63. EKM80065
64. EKM81586
65. EKM81594
66. EKM84339
67. EMD31385
68. EMD31470
69. EMD34893 ----------------RAARRSFCPARS----LWF
70. EMD36590
71. EMD37041
72. EMD37069
73. EMD37778
74. EMD37997
75. EMD38969
76. EMD39451
77. EMD40118
78. EMD40904
79. EPQ62657 ----EKLEGQSEKLEEQAEKLEEQPEKLEGQSEKLEDEAEK----------LEEQPEKLEDEAEDNIEGESKKEDTSVEKLDGSNEDDDDDDDDEDDEDDEDDEDSASAPIANPPGTKTSLKAPLNG
80. EPQ63654
81. EPQ66893
82. EUC33016
83. EUC34114
84. EUC36558
85. FGRAMPH1_01T01083
86. FGRAMPH1_01T01333
87. FOXG_00537P0
88. FOXG_00636P0
89. FVEG_00876T0
90. FVEG_00977T0
91. KDB12319
92. KDB15881
93. KIS67867
94. KIS70693
95. KIS72283
96. KLO94325 --MLFQIKRQDRRRDTQ
97. KNZ46184
98. KNZ50279
99. KNZ52281
100. KNZ55116
101. KNZ55117
102. KNZ55822 ---------------TTCSRSPHNRRVAKVLRT
103. KUI53186
104. KUI55655
105. KUI57242
106. KUI58831
107. KUI59152
108. KUI59516
109. KUI59652
110. KUI61379 ETAVLLVDYQNIHVQHAGPKGMEAITIAQSLRKWADEHRIPVIIARTDTSQVPPRHAKLHDRAIDILKAFEATP-----------------------------GLGAVHPALTPPKP---ASQPVHD
111. MGG_00527T0
112. MGG_04599T0
113. MGG_09641T0
114. MGG_09736T0
115. Mycgr3P72251
116. Mycgr3P78594
117. Mycgr3P102016
118. Mycgr3P106456
119. Mycgr3P107242
120. NechaP87001 --LFFVMRRRKQKAAKGSTSSADPRDGQPQIAELEQPKNINIVVTLATPAKIRI
121. NechaP98847
122. NechaP102374
123. VDAG_JR2_Chr3g01910a-00001
124. VDAG_JR2_Chr3g03370a-00001

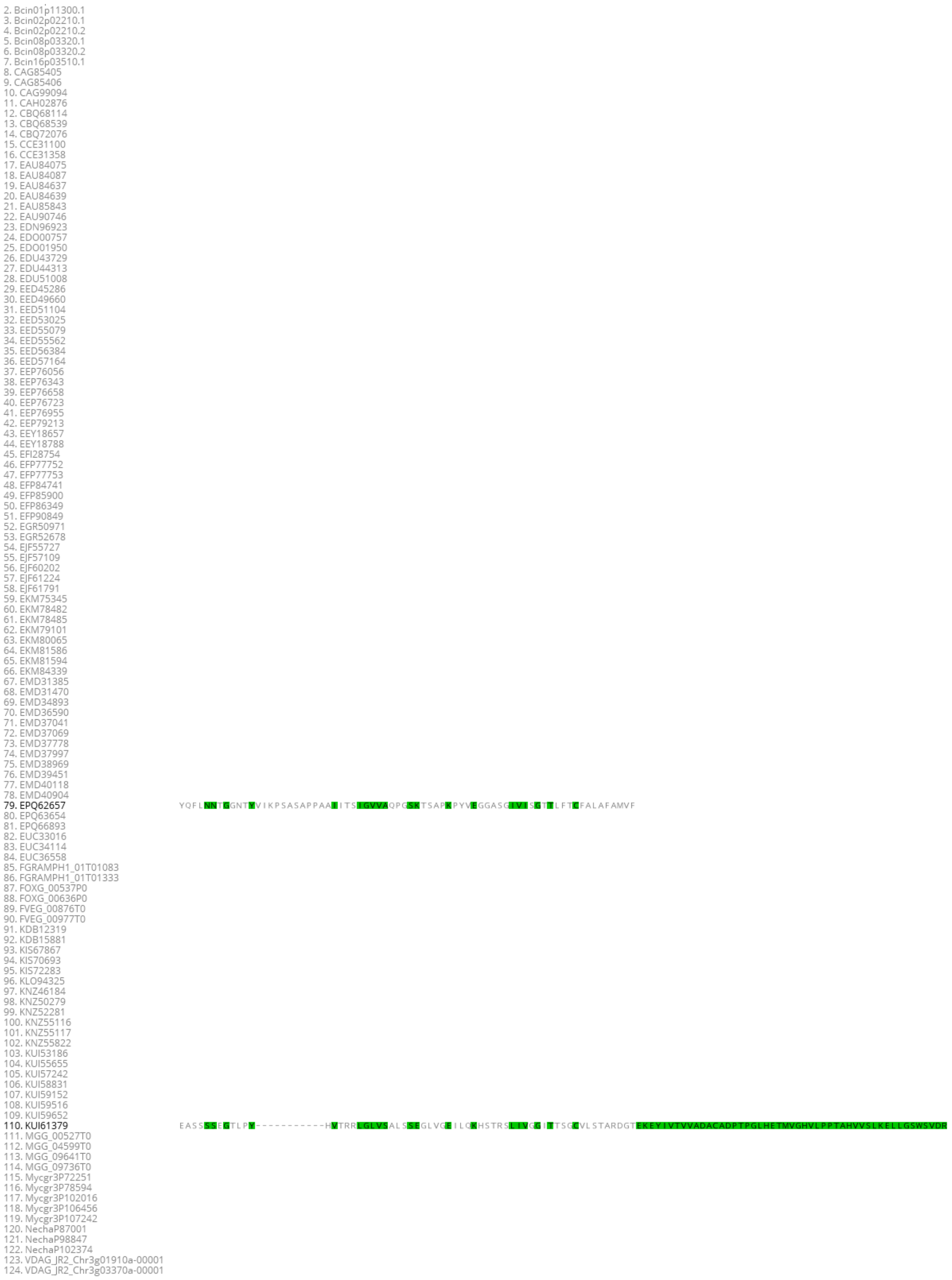

Supplementary Fig. 5 ClustalW alignment of 124 protein sequences that contain the Ser-Thr-rich Glycosyl-phosphatidyl-inositol-anchored domain. The protein sequences and related information are provided in Supplementary Data 2

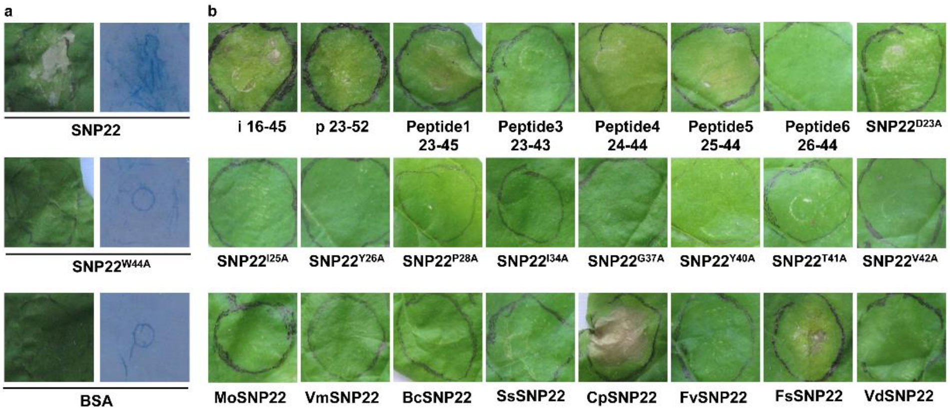

Supplementary Fig. 6 Cell death responses induced by synthesized peptides. a, Cell death responses induced by synthesized 10 μM SNP22 or 10 μM SNP22^{W44A}. Left, representative cell death phenotypes in *N. benthamiana* leaves photographed at 5 dpi. Right, trypan blue staining. **b,** Cell death responses induced by other synthesized peptides. Photos were taken at 5 dpi. *Bc*, Bcin01p05310.2 (*Botrytis cinerea*); *Ss*, EDO01950 (*Sclerotinia sclerotiorum*); *Mo*, MGG_04599T0 (*Magnaporthe oryzae*); *Vm*, KUI59516 (*Valsa mali*); *Vd*, VDAG_JR2_Chr3g03370a-00001 (*Verticillium dahliae*); *Fs*, NechaP98847 (*Fusarium solani*); *Fv*, FVEG_00876T0 (*Fusarium verticillioides*); *Cp*, CCE31100 (*Claviceps purpurea*). Three independent experiments were performed as shown in Supplementary Data 3. For each experiment, each peptide was infiltrated into 10 different plants.

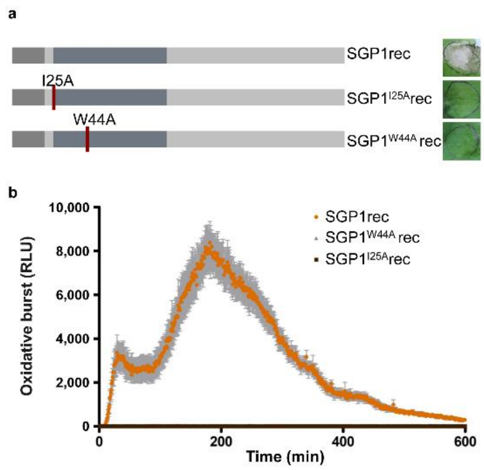

Supplementary Fig. 7 The I25A and W44A mutations attenuate the ability of SGP1 to induce cell death and ROS production. a, Cell death triggered by SGP1^{I25A}rec and SGP1^{W44A}rec in *N. benthamiana*. **b,** ROS accumulation induced by SGP1rec and SGP1 mutants in *N. benthamiana* leaf discs. Leaf discs from 3- to 4-week-old *N. benthamiana* plants were incubated with the indicated recombinant proteins at a concentration of 1 μM. Luminescence is expressed as relative light units (RLU). Values represent means ± SEM of three leaf disks, each sampled from a different plant and tested in parallel.

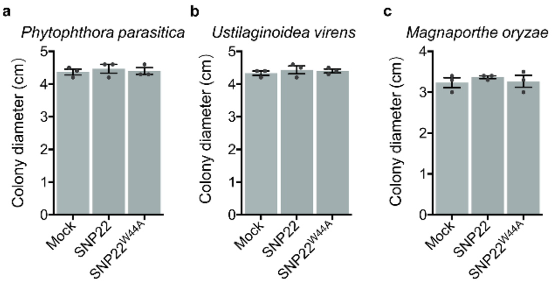

Supplementary Fig. 8 The synthesized peptides do not affect filamentous growth of pathogens on plates. a, Colony diameter of *P. parasitica* on V8 medium containing the indicated peptides. Data were collected after 3 days of growth. **b,** Colony diameter of *U. virens* on PSA medium containing the indicated peptides. Data were collected after 14 days of growth. **c,** Colony diameter of *M. oryzae* on PSA medium containing the peptides. Data were collected after 7 days of growth. Means ± SEM of three independent experiments are shown.

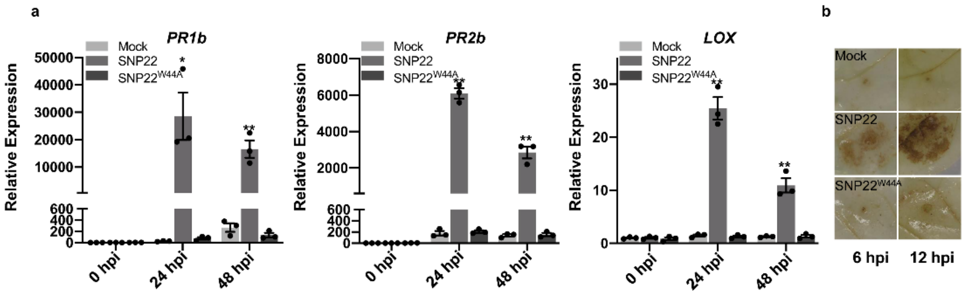

Supplementary Fig. 9 Activation of immunity in *N. benthamiana* triggered by peptides. a, Relative expression of PR genes in *N. benthamiana* leaves after treatment with 1 μM SNP22 or SNP22^{W44A} peptides. The relative transcript levels of PR genes were measured by qRT-PCR and normalized to those of the mock treatment using the *NbEF1α* gene as an internal reference. Values are the means ± SEM of three biological replicates (*, P<0.05, **, P<0.01, compared with Mock, two-sided Dunnett's test). **b,** DAB staining of *N. benthamiana* leaves treated by SNP22. *N. benthamiana* leaves were infiltrated with 1 μM of the indicated peptides 6 h or 12 h before DAB staining.

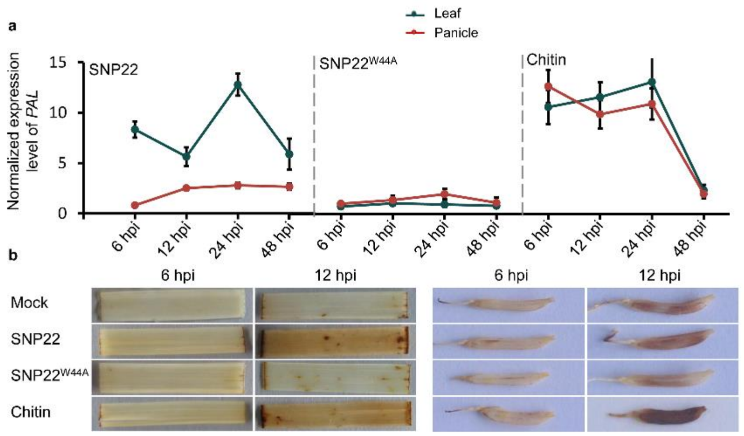

Supplementary Fig. 10 Activation of immunity in rice triggered by elicitors. a, Relative induction of defense-related gene expression in rice leaves and panicles triggered by the indicated elicitors. Rice leaves and panicles were treated with 1 μM SNP22 peptide, SNP22^{W44A} peptide, or chitin. Defense-related gene expression was normalized to that of the mock treatment at each time point after treatment, with rice *actin* as an internal reference. Values represent the means ± SEM of three biological replicates. **b,** DAB staining of rice leaves and panicles treated with elicitors.

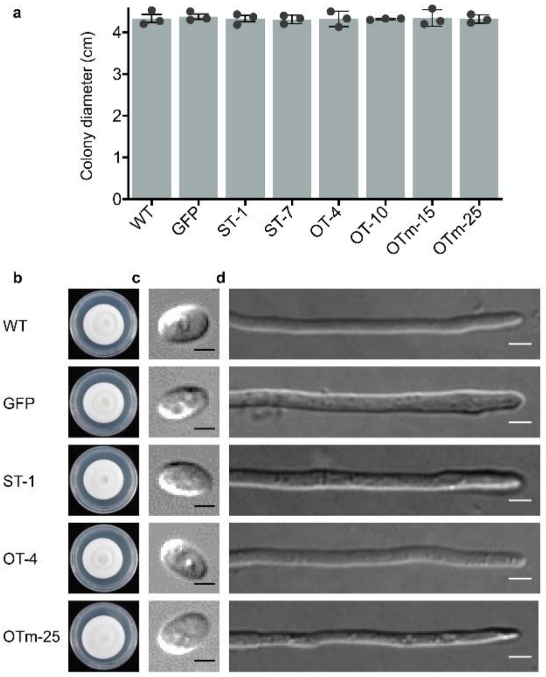

Supplementary Fig. 11 Silencing of SGP1 or overexpression of SGP1 or SGP1^{W44A} does not affect *U. virens* filamentous growth, conidia morphology, or hyphal morphology. a, Colony diameter of different *U. virens* transgenic lines. Values are means ± SEM of three independent experiments. In each experiment, colonies on three plates were measured. **b,** Representative images of filamentous growth of different *U. virens* transgenic lines. Photographs were taken after 14 days of growth on PSA medium. **c,** Representative images of conidia of different *U. virens* transgenic lines. Bar = 2.5 μm. **d,** Representative images of mycelia of different *U. virens* transgenic lines. Bar = 4 μm.
