# Supplementary material for: The N-terminus of an Ustilaginoidea virens Ser-Thr-rich glycosylphosphatidylinositol-anchored protein elicits plant immunity as a MAMP
Source: Nat Commun. 2021 Apr 27;12:2451. doi: 10.1038/s41467-021-22660-9 (PMC8079714; doi:10.1038/s41467-021-22660-9)
Supplement: Supplementary file 2 — Descriptions of Additional Supplementary Files [file 41467_2021_22660_MOESM2_ESM.pdf]

Descriptions of Additional Supplementary Files

Supplementary Data 1
Description: List of all PAMP candidate genes from the secreted proteins of *Ustilaginoidea virens*.

Supplementary Data 2
Description: List of proteins containing a Ser-Thr-rich Glycosyl-phosphatidyl-inositol-anchored domain in fungal species.

Supplementary Data 3
Description: Statistics of all cell death phenotypes.

Supplementary Data 4
Description: Fasta files for sequence alignment of 124 sequences in Supplementary Fig. 5.

Supplementary Data 5
Description: Single copy orthologous proteins in 30 species identified by OrthoFinder.

Supplementary Data 6
Description: Sequences used for construction of the sequence logos of the 22 amino acids shown in Fig. 4d.

Supplementary Data 7
Description: Sequences used for expression of recombinant proteins and cell death assays in *N. benthamiana* shown in Fig. 4e.

Supplementary Data 8
Description: Oligonucleotides used in the study.

Supplementary Data 9
Description: Constructs and construction methods used in this study.

Supplementary Data 10
Description: Number of mapped reads.
