# Supplementary material for: The N-terminus of an Ustilaginoidea virens Ser-Thr-rich glycosylphosphatidylinositol-anchored protein elicits plant immunity as a MAMP
Source: Nat Commun. 2021 Apr 27;12:2451. doi: 10.1038/s41467-021-22660-9 (PMC8079714; doi:10.1038/s41467-021-22660-9)
Supplement: Supplementary file 13 — Reporting Summary [file 41467_2021_22660_MOESM13_ESM.pdf]

nature research

Corresponding author(s): Yongfeng Liu

Last updated by author(s): Mar 12, 2021

Reporting Summary

Nature Research wishes to improve the reproducibility of the work that we publish. This form provides structure for consistency and transparency in reporting. For further information on Nature Research policies, see our Editorial Policies and the Editorial Policy Checklist.

Statistics

For all statistical analyses, confirm that the following items are present in the figure legend, table legend, main text, or Methods section.

n/a	Confirmed	
☐	☒	The exact sample size (*n*) for each experimental group/condition, given as a discrete number and unit of measurement
☐	☒	A statement on whether measurements were taken from distinct samples or whether the same sample was measured repeatedly
☐	☒	The statistical test(s) used AND whether they are one- or two-sided<br>*Only common tests should be described solely by name; describe more complex techniques in the Methods section.*
☒	☐	A description of all covariates tested
☒	☐	A description of any assumptions or corrections, such as tests of normality and adjustment for multiple comparisons
☐	☒	A full description of the statistical parameters including central tendency (e.g. means) or other basic estimates (e.g. regression coefficient) AND variation (e.g. standard deviation) or associated estimates of uncertainty (e.g. confidence intervals)
☐	☒	For null hypothesis testing, the test statistic (e.g. *F*, *t*, *r*) with confidence intervals, effect sizes, degrees of freedom and *P* value noted<br>*Give P values as exact values whenever suitable.*
☒	☐	For Bayesian analysis, information on the choice of priors and Markov chain Monte Carlo settings
☒	☐	For hierarchical and complex designs, identification of the appropriate level for tests and full reporting of outcomes
☒	☐	Estimates of effect sizes (e.g. Cohen's *d*, Pearson's *r*), indicating how they were calculated

Our web collection on statistics for biologists contains articles on many of the points above.

Software and code

Policy information about availability of computer code

Data collection	no software was used
Data analysis	MEGA6, OrthoFinder v 2.2.6,PhyML v3.0,ClustalW2 program, TopHat version 2.1.1, and Geneious 11 were used for sequece analysis; SPSS Statistics 19 and GraphPadPrism 9 were used for data analysis; seaborn 0.11.1 was used to generate box-plot (https://seaborn.pydata.org/index.html);Cufflinks version 2.2.1 were used for RNA-seq data analysis; Proteome Discoverer 2.1 were used for LC-MS/MS data analysis.

For manuscripts utilizing custom algorithms or software that are central to the research but not yet described in published literature, software must be made available to editors and reviewers. We strongly encourage code deposition in a community repository (e.g. GitHub). See the Nature Research guidelines for submitting code & software for further information.

Data

Policy information about availability of data

All manuscripts must include a data availability statement. This statement should provide the following information, where applicable:

- Accession codes, unique identifiers, or web links for publicly available datasets
- A list of figures that have associated raw data
- A description of any restrictions on data availability

The data that support the findings of this study are available in the manuscript and its supplementary files or are available from the corresponding authors upon reasonable request. RNA-seq data generated in this study are accessible in the NCBI Sequence Read Archive data repository under the accession number PRJNA690691(https://www.ncbi.nlm.nih.gov/sra/?term=PRJNA690691). Proteomics data in this study are accessible in Proteome Exchange partner repository under the accession number PXD02454 (https://www.ebi.ac.uk/pride/archive). The data underlying Figs 1d-e, 2h, 3b-c, 4b, 5b-d, 6b-c, 6e-f, 6h-I, 7a-c, 7f, 7h, 8a, 8c, 8e, 8f-g, Supplementary Figs. 3, 7b, 8, 9a, 10a, 11a, and Supplementary Table. 2 are provided as a Source Data file.

Field-specific reporting

Please select the one below that is the best fit for your research. If you are not sure, read the appropriate sections before making your selection.

[x] Life sciences [ ] Behavioural & social sciences [ ] Ecological, evolutionary & environmental sciences

For a reference copy of the document with all sections, see nature.com/documents/nr-reporting-summary-flat.pdf

Life sciences study design

All studies must disclose on these points even when the disclosure is negative.

Sample size	Sample sizes were determined based on the generation of convincing and compelling results. At least three biological replicates were performed in all experiments. The number of replicates is indicated in the figure legend and/or in the method section.
Data exclusions	No data were excluded unless the experiment failed. For example, if injection of the inoculum into the swollen sheaths failed, the panicle was not counted.
Replication	Experiments were repeated at least three times with similar results.
Randomization	Samples were allocated randomly into experimental groups.
Blinding	Investigators were blinded to the cell death- and pathogenicity-related assays by labeling each strain or treatment with simple, non-informative labels and randomized allocation of mock and infected plants in the greenhouse. Investigators were not blinded to western blots because the investigators needed to determine appropriate samples to run in particular lanes of the gel.

Reporting for specific materials, systems and methods

We require information from authors about some types of materials, experimental systems and methods used in many studies. Here, indicate whether each material, system or method listed is relevant to your study. If you are not sure if a list item applies to your research, read the appropriate section before selecting a response.

Materials & experimental systems

n/a	Involved in the study	
[ ]	[x]	Antibodies
[x]	[ ]	Eukaryotic cell lines
[x]	[ ]	Palaeontology and archaeology
[x]	[ ]	Animals and other organisms
[x]	[ ]	Human research participants
[x]	[ ]	Clinical data
[x]	[ ]	Dual use research of concern

Methods

n/a	Involved in the study	
[x]	[ ]	ChIP-seq
[x]	[ ]	Flow cytometry
[x]	[ ]	MRI-based neuroimaging

Antibodies

Antibodies used	Anti-HA-tag primary monoclonal antibody (M20003L, Abmart,1:3000); Anti-GFP-tag primary monoclonal antibody (M20004L, Abmart,1:3000); HRP-conjugated Anti- HA antibodies (26183-HRP, Thermo scientific, 1:2000); IRDye 800CW Goat anti-Mouse IgG(H+L) antibody (926-32210, LI-COR,1:10000); Anti-phospho-p44/42 MAPK (Erk1/2) antibody (#4370s, Cell Signaling,1:1000) and IRDye 800CW Goat anti-Rabbit IgG(H+L) antibody (926-32211, LI-COR,1:10000)
Validation	All antibodies recognize tagged proteins, though unspecific bands were also detected. Anti-HA-tag primary monoclonal antibody was used to validate the expression of tagged protein in N. benthamiana; Anti-GFP-tag primary monoclonal antibody was used to validate the expression of SGP1 and its mutants in U. virens. Anti-phospho-p44/42 MAPK (Erk1/2) antibody was used to validate the phosphorylation of MAPK proteins in rice.
